# TYK2 inhibition reduces type 3 immunity and modifies disease progression in murine spondyloarthritis

**DOI:** 10.1172/JCI126567

**Published:** 2020-03-09

**Authors:** Eric Gracey, Dominika Hromadová, Melissa Lim, Zoya Qaiyum, Michael Zeng, Yuchen Yao, Archita Srinath, Yuriy Baglaenko, Natalia Yeremenko, William Westlin, Craig Masse, Mathias Müller, Birgit Strobl, Wenyan Miao, Robert D. Inman

**Affiliations:** 1 Department of Immunology, University of Toronto, Toronto, Ontario, Canada.; 2 Genetics and Development, Krembil Research Institute, University Health Network, Toronto, Ontario, Canada.; 3 Spondylitis Program, Toronto Western Hospital, University Health Network, Toronto, Ontario, Canada.; 4 Institute of Animal Breeding and Genetics, University of Veterinary Medicine, Vienna, Austria.; 5 Divisions of Genetics and Rheumatology, Department of Medicine, Brigham and Women’s Hospital and Harvard Medical School, Boston, Massachusetts, USA.; 6 Division of Clinical Immunology and Rheumatology, Department of Experimental Immunology, Academic Medical Center, Amsterdam, Netherlands.; 7 Nimbus Therapeutics, Cambridge, Massachusetts, USA.

**Keywords:** Autoimmunity, Therapeutics, Arthritis, Cytokines, T cells

## Abstract

Spondyloarthritis (SpA) represents a family of inflammatory diseases of the spine and peripheral joints. Ankylosing spondylitis (AS) is the prototypic form of SpA in which progressive disease can lead to fusion of the spine. Therapeutically, knowledge of type 3 immunity has translated into the development of IL-23– and IL-17A–blocking antibodies for the treatment of SpA. Despite being able to provide symptomatic control, the current biologics do not prevent the fusion of joints in AS patients. Thus, there is an unmet need for disease-modifying drugs. Genetic studies have linked the Janus kinase TYK2 to AS. TYK2 is a mediator of type 3 immunity through intracellular signaling of IL-23. Here, we describe and characterize a potentially novel small-molecule inhibitor of TYK2 that blocked IL-23 signaling in vitro and inhibited disease progression in animal models of SpA. The effect of the inhibitor appears to be TYK2 specific, using TYK2-inactive mice, which further revealed a duality in the induction of IL-17A and IL-22 by IL-23. Specifically, IL-22 production was TYK2/JAK2/STAT3 dependent, while IL-17A was mostly JAK2 dependent. Finally, we examined the effects of AS-associated *TYK2* SNPs on *TYK2* expression and function and correlated them with AS disease progression. This work provides evidence that TYK2 inhibitors have great potential as an orally delivered therapeutic for SpA.

## Graphical abstract

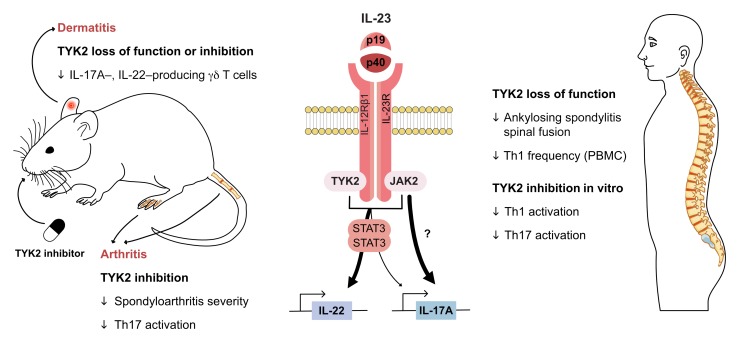

## Introduction

The spondyloarthritis (SpA) family of inflammatory joint diseases are united by the presence of axial or peripheral arthritis without the presence of defined autoantibodies, young age of onset, and entheseal origin of inflammation (1, 2). Clinically, SpA commonly coexists with gut and skin inflammation in the form of inflammatory bowel disease (IBD) and psoriasis, respectively. The natural history of SpA is characterized by chronic inflammation, followed by sequential bone erosion and new bone formation. Patients with late-stage disease, especially males, can develop fused joints, which results in reduced mobility and considerable loss in quality of life (3).

Ankylosing spondylitis (AS) and psoriatic arthritis (PsA) are the major forms of SpA. Clinically, AS is closely linked with IBD, especially Crohn’s disease, and arthritis is primarily axial by diagnostic definition (4). AS may also involve peripheral arthritis and psoriasis (1). PsA, on the other hand, is closely linked with psoriasis according to its classification criteria (5), and the arthritis tends to be peripheral but can include an axial component (6).

Immunologically, SpA is typified by systemic overactivity of type 3 immunity (7), the effector arm of the immune system that controls extracellular bacteria and fungi (8). The conventional view is that IL-23 strengthens type 3 immunity by upregulating IL-17A and IL-22 in RORγt^+^ lymphocytes. Many IL-17A–producing cell types have been implicated in SpA, including Th17 cells and γδ T cells (9).

Therapeutically, knowledge of type 3 immunity has translated to the development of IL-23– and IL-17A–blocking antibodies (biologics) for the treatment of SpA. These new biologics are generally as effective as TNF inhibitors at providing symptomatic control of SpA (10, 11); however, the effectiveness of these therapeutics differs in patients with PsA versus AS: despite being able to modify the progression of joint damage in PsA, the current biologics have not been conclusively shown to prevent the fusion of joints in AS patients. Recent long-term follow-up studies have shown that TNF inhibitors may slow, but do not stop, radiographic progression in AS (12). Anti–IL-17A agents may have a similar effect, but longer follow up studies are needed (13). Thus there is an urgent unmet need for truly disease-modifying drugs for AS patients that will prevent the inexorable progression of spinal ankylosis.

One approach in the hunt for novel therapeutic targets for AS is to focus on factors genetically linked to the disease. A number of type 3 immunity–related genes have been linked to SpA through genome-wide association studies (GWAS), such as *IL23R*, *IL6R*, *IL12B*, *JAK2*, and *TYK2* (14). *JAK2* and *TYK2*, both coding for Janus kinases (JAKs), are of particular interest given the large number of orally available small-molecule inhibitors developed recently (15). JAK inhibitors (JAKinibs) already are approved for clinical use in rheumatoid arthritis and PsA, and a recent proof-of-concept trial showed that the pan-JAK inhibitor tofacitinib provided both symptomatic control in AS and reduced axial joint inflammation on MRI (16). It is therefore plausible that JAKinibs could fill the unmet need as disease-modifying therapeutics in AS.

TYK2 mediates signaling through the type 1 IFN receptor (IFNAR), IL-10 family receptors (IL-10R and IL-22R), and IL-12 family receptors (IL-12R and IL-23R). Humans naturally deficient in TYK2 have increased sensitivity to mycobacterial and viral infections (17, 18), while TYK2-deficient mice are protected from Th17-mediated autoimmune disease (19–21). To date, a number of single-nucleotide polymorphisms (SNPs) around the *TYK2* locus (22–24) have been associated with AS (Supplemental Figure 1; supplemental material available online with this article; https://doi.org/10.1172/JCI126567DS1). It is worth noting that exonic *TYK2* SNPs associated with AS are coding variants that have been previously shown to be associated with a loss of function (LoF) both ex vivo and in vitro (23, 25, 26). These LoF SNPs are shared with related inflammatory diseases, including PsA and Crohn’s disease (23, 27, 28). Indeed rs34536443 (P1104A), one of the primary *TYK2* LoF SNPs associated with multiple autoimmune diseases, is protective against disease, but does not impact on nonautoimmune traits such as susceptibility to infection (23, 29). These data support the concept of therapeutic targeting of TYK2 without associated immunosuppression.

Here we characterize a novel, orally bioavailable small-molecule inhibitor of TYK2 that is effective at inhibiting IL-23 signaling in vitro and is effective at inhibiting SpA progression in murine models. We confirm that the effect of the inhibitor is TYK2-specific, using TYK2-inactive mice, which revealed a duality in the induction of IL-17A and IL-22 by IL-23. Finally, we address the biological effects of AS-associated *TYK2* SNPs, revealing a possible role for TYK2 in spinal fusion.

## Results

### NDI-031407 is a potent and selective TYK2 inhibitor.

**Figure 1:**
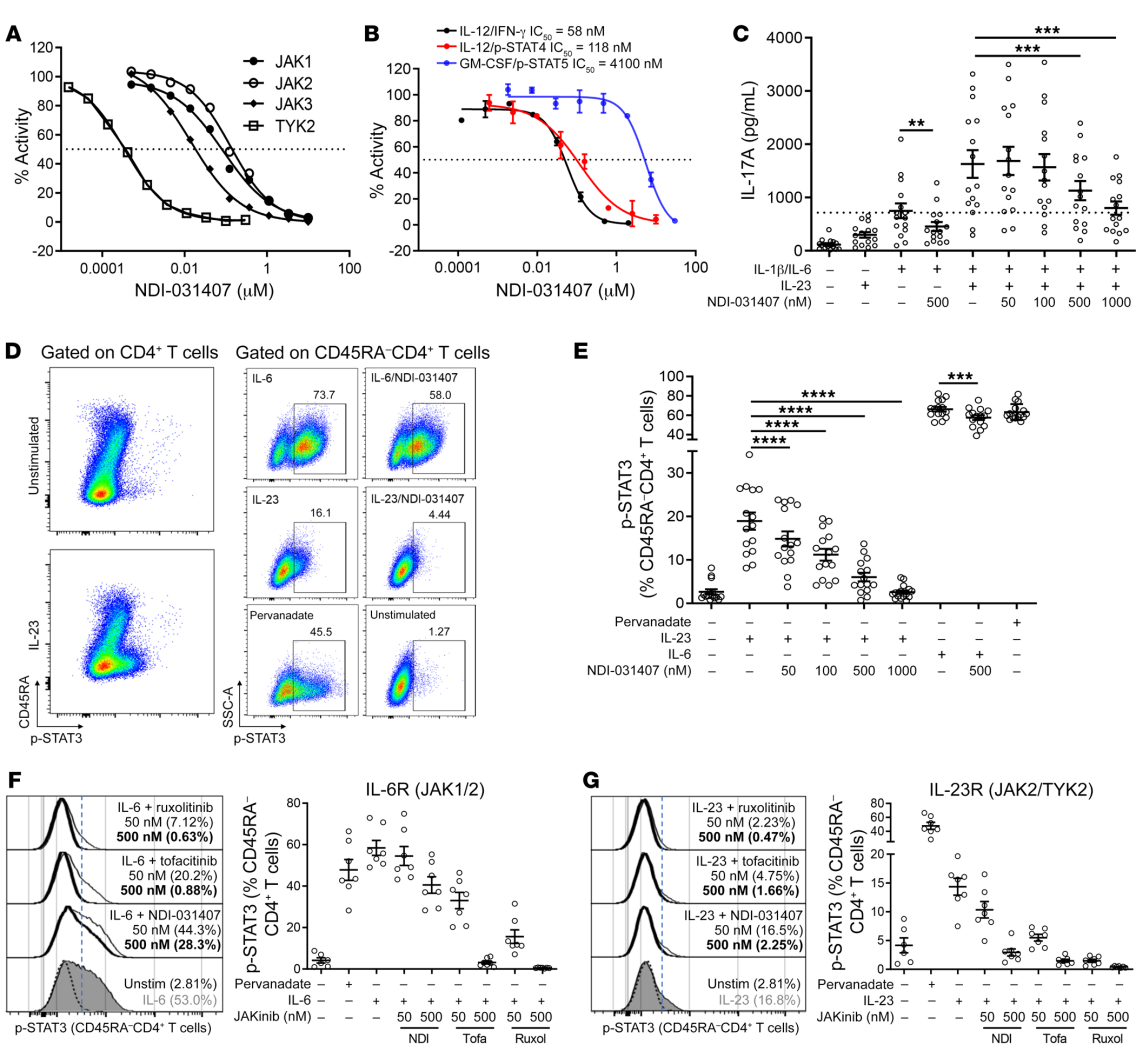
TYK2 inhibition by a novel small molecule blocks IL-23–induced STAT3 phosphorylation and IL-17A production in human CD4^+^ T cells. (**A** and **B**) NDI-031407, a novel TYK2 inhibitor, was tested for: (**A**) Specificity for TYK2 against JAK1–3 kinases by radiometric assay with peptide substrates. Activity represents the ratio of activated substrate in DMSO versus inhibitor treatment. (**B**) Potency for IL-12–induced p-STAT4 and GM-CSF–induced p-STAT5 in PMBCs and IL-12–induced IFN-γ in NK92 cells. Activity represents the ratio of p-STAT to total STAT. Data in **A** and **B** are from a single experiment, representative of 3 independent experiments. The horizontal lines represent 50% inhibition. (**C**) Magnetically purified CD4^+^ T cells were cultured with anti-CD2/CD3/CD28 beads for 3 days with NDI-031407 in the presence of 20 ng/mL of cytokines. At endpoint, IL-17A was assessed in the culture supernatant by ELISA. (**D**–**G**) PBMCs were stimulated for 4 days with anti-CD2/CD3/CD28 beads. Cells were then serum-starved and pretreated with JAKinib for 30 minutes before 15-minute stimulation with pervanadate, 400 ng/mL IL-6, or 400 ng/mL IL-23. STAT phosphorylation was assessed by flow cytometry. (**D**) Representative dot plots showing p-STAT3 in relation to mature CD4^+^ T cells (left) and representative gating for p-STAT3^+^ cells in mature CD4^+^ T cells with the indicated treatments (right). (**E**) Pooled data showing p-STAT3 in mature CD4^+^ T cells. (**F** and **G**) Comparison of NDI-031407, tofacitinib, and ruxolitinib inhibition of IL-23R and IL-6R. Representative histograms show p-STAT3 in mature CD4^+^ T cells: unstimulated (black dashed line), cytokine-stimulated (gray shading), or 50 nM (thin lines) and 500 nM (thick lines) of the respective JAKinib. Threshold used to gate p-STAT3^+^ (blue dashed line) and percentage positive are indicated in parentheses. Graph title indicates the cytokine-associated JAKs. (**C** and **E**) IL-6/vehicle vs. IL-6/500 nM NDI-031407 by Wilcoxon matched-pairs signed-rank test and stimulated/vehicle-treated wells vs. stimulated/NDI-031407–treated wells by paired 1-way ANOVA with Dunnett’s post hoc test comparing treatments with vehicle control. For all scatter plots, each point represents an independent donor. **P < 0.01, ***P < 0.001, ****P < 0.0001.

Nimbus Therapeutics has developed novel small-molecule inhibitors of TYK2. NDI-031407 is an inhibitor of the catalytic (kinase) domain of TYK2 and has high selectivity over other JAK family members. Specifically, the average IC_50_ for TYK2 inhibition in radiometric assays was 0.21 nM, which was 20 times more effective at inhibiting JAK3 (4.2 nM), 147 times for JAK2 (31 nM), and 220 times for JAK1 (46 nM) (Figure 1A). Further, NDI-031407 has high potency in cell line and primary cell assays for TYK2-dependent cytokines (IL-12) over TYK2-independent cytokines (GM-CSF) (Figure 1B). Given the genetic link of AS to the IL-23/IL-17 pathway and the role of TYK2 in IL-23 signaling, we first aimed to examine the effects of NDI-031407 on human Th17 cells in vitro.

As freshly isolated PBMCs are refractory to IL-23 stimulation, cell activation is required. We followed a protocol in which magnetically sorted CD4^+^ T cells are skewed to produce IL-17A with TCR and cytokine stimulation (30). In this 3-day assay, IL-23 synergized with IL-1β/IL-6 to enhance IL-17A production, which was dose-dependently inhibited by NDI-031407 (Figure 1C). In this assay, NDI-031407 had no effect on apoptosis and had a protective effect against cell death (Supplemental Figure 2). NDI-031407 did limit, but did not inhibit, cell proliferation. These results were comparable to effects seen with tofacitinib.

Given the effect of NDI-031407 on IL-23–induced IL-17A, we explored the role of TYK2 in IL-23–induced STAT3 phosphorylation. TCR-stimulated PBMCs were rested in serum-free media before a 15-minute stimulation with IL-23. NDI-031407 dose-dependently inhibited IL-23–induced phosphorylation of STAT3 (p-STAT3) in mature CD4^+^ T cells (Figure 1, D and E), demonstrating the critical role of TYK2 in IL-23 signaling in activated T cells. The gating strategy and IL-23–specific activation of STAT3 over STAT4 and STAT5 are illustrated in Supplemental Figure 3.

To compare the selectivity of NDI-031407 for TYK2 with clinically approved JAKinibs, we characterized p-STAT3 in a separate cohort of subjects. As expected, tofacitinib and ruxolitinib, both pan-JAK inhibitors, were more effective than NDI-031407 at blocking JAK1/JAK2-dependent IL-6 signaling (Figure 1F). While both tofacitinib and ruxolitinib were able to block IL-23–induced STAT3 phosphorylation, likely by inhibiting JAK2 and to a lesser extent TYK2, NDI-031407 was equally effective at the higher dose tested (Figure 1G). NDI-031407 is thus effective at blocking TYK2-dependent signaling in Th17 cells in vitro, and represented a promising lead for therapeutic testing in vivo.

### NDI-031407 inhibits disease progression in the SKG model of SpA.

**Figure 2:**
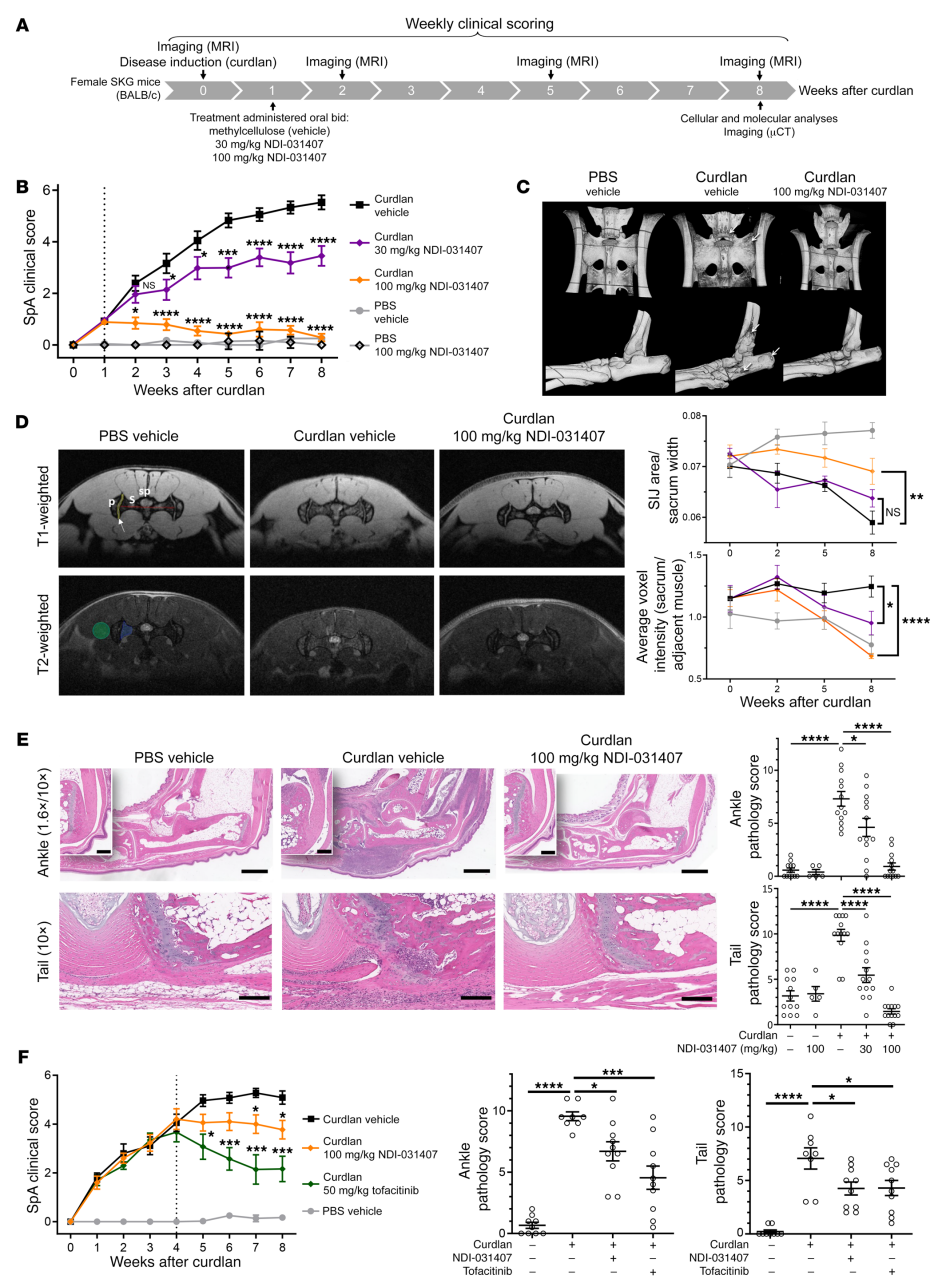
TYK2 inhibition by small molecule prevents SpA disease progression in SKG mice. Female SKG mice were treated with curdlan to induce SpA-like disease, or with PBS as disease-free controls. (**A**) Overview of experiment and readouts for **B**–**E**. At 1 week after curdlan treatment, mice began treatment with NDI-031407 at the indicated dosages by gavage twice daily. (**B**) Mice (*n* = 12–13 per group) were scored weekly for SpA symptoms (blepharitis, arthritis, and dermatitis). (**C**) Representative postmortem μCT images of pelvis (dorsal view) and ankles (lateral view) from mice in the indicated groups. Arrows point to sites of entheseal erosion. (**D**) MRI of the sacroiliac joint (SIJ; coronal plane) in live mice. T1 weighing was used to assess SIJ area as a ratio to sacrum width. SP, spinal cord; S, sacrum; P, pelvis; red line, sacrum width; yellow area/white arrow, SIJ space. Scale bar: 5 mm. T2 weighing was used to assess bone marrow edema in the sacrum (blue area) normalized to adjacent muscle (green area). Representative images at 8 weeks after disease induction. Pooled data from 5 mice per group. Group colors are the same as in **B**. (**E**) H&E staining and scoring of tissue at 8 weeks after curdlan. Scale bars: 1 mm for 1.6× and 200 μm for 10×. (**F**) SKG mice were treated from 4 weeks after curdlan (therapeutically) with NDI-031407 or tofacitinib for 4 weeks (*n* = 9–10 per group). Data in **B**, **D**, and **F** were assessed by 2-way ANOVA, with time considered as dependent variable. Means of curdlan/NDI-031407–treated animals compared with curdlan/vehicle-treated controls at each time point by Dunnett’s post hoc test. For pathology scoring (**E** and **F**), each point represents a single mouse; disease-free vs. curdlan/vehicle animals were analyzed by unpaired *t* test, NDI-031407–treated vs. vehicle-treated animals by 1-way ANOVA with Dunnett’s post hoc test. *P < 0.05, **P < 0.01, ***P < 0.001, ****P < 0.0001.

We first tested the therapeutic efficacy of NDI-031407 in SKG mice, an IL-23– and Th17-dependent model of SpA (31). In this model, BALB/c mice with a hypomorphic ZAP70 remain disease-free under specific pathogen–free conditions. A single dose of the dectin agonist curdlan induces progressive SpA-like disease that is observed experimentally over the course of 8 weeks (31, 32). An experimental overview is given in Figure 2A.

We initiated oral twice-daily treatment with NDI-031407 at 1 week after curdlan treatment, to allow for disease establishment. Scoring of clinical symptoms showed a dose response with NDI-031407, with the highest dose completely blocking disease progression (Figure 2B).

X-ray evidence of axial skeleton bone modification is the gold standard for diagnosis of AS in the clinical setting. We performed postmortem, high-resolution micro-CT (μCT) of target bony structures. This imaging revealed erosion in the axial and peripheral skeleton (Figure 2C and Supplemental Figure 4A). More specifically, these erosions were evident at points of sacroiliac and vertebral ligament insertions in the pelvis and tail. In the ankle, the erosions were seen around Achilles tendon insertion on the calcaneus, and at entheses where the peroneus longus and brevis insert (33). These erosions were absent in SKG mice treated with the high dose of NDI-031407. MRI of the sacroiliac joint (SIJ) plays an important role for early diagnosis of AS owing to its ability to detect inflammatory changes prior to radiographic changes. We therefore performed MRI to assess joint space narrowing and bone marrow edema by T1 and T2 weighting respectively (Figure 2D). Consistent with readouts in humans, disease progression in the SKG mice was reflected by joint space narrowing and bone marrow edema, which was prevented with NDI-031407 treatment.

Histopathology was used to confirm imaging observations in the SKG experiments. In our hands, the onset of small intestine inflammation (enteritis) in the SKG model was sporadic (Supplemental Figure 4, B and C), which confounded analysis of this tissue. In the pelvis and sacrum, the density of bone marrow leukocytes was increased, supportive of MRI observations of edema; however, overall pathology scores of the SIJ did not differ drastically with disease (Supplemental Figure 4, D and E). This is likely due to the coronal plane of our sections missing the dorsal and ventral erosions at SIJ ligament insertion seen with μCT. In the ankle and tail, an inflammatory infiltrate was seen around tendons and ligaments (Figure 2E), consistent with the entheseal origin of SpA in these tissues (34). Bone erosions occurred at sites adjacent to the entheses, consistent with our μCT imaging observations and the bone marrow edema seen by MRI. Scoring of ankle and tail pathology illustrated a dose-dependent, protective response with NDI-031407 (Figure 2E).

Finally, we performed therapeutic dosing experiments with treatment starting at 4 weeks, when arthritis is fulminant (Figure 2F). For this set of experiments, we included tofacitinib as a control, at a dose commonly reported in the literature (35). There was a reduction in clinical scoring with NDI-031407 treatment, albeit predictably not as strong as in those with tofacitinib treatment. Histopathological examination of the ankles and tails revealed a significant reduction in pathology with both inhibitors. In sum, these data suggest a role for TYK2 in disease progression, which prompted us to examine its effects on type 3 immune cells in vivo.

### NDI-031407 controls Th17 cell frequency and SpA in SKG mice.

Activation of autoreactive Th17 cells is central to immunopathogenesis in the SKG mouse model (36). We therefore examined lymph nodes draining gut, peripheral, and axial joints, namely the mesenteric (MLNs), popliteal (PLNs), and sciatic lymph nodes (SLNs), respectively. Cell counts showed lymphadenopathy of the joint-associated, but not the gut-associated, lymph nodes (Supplemental Figure 5A), which was consistent with inflammation seen by histopathology of the corresponding tissues.

**Figure 3:**
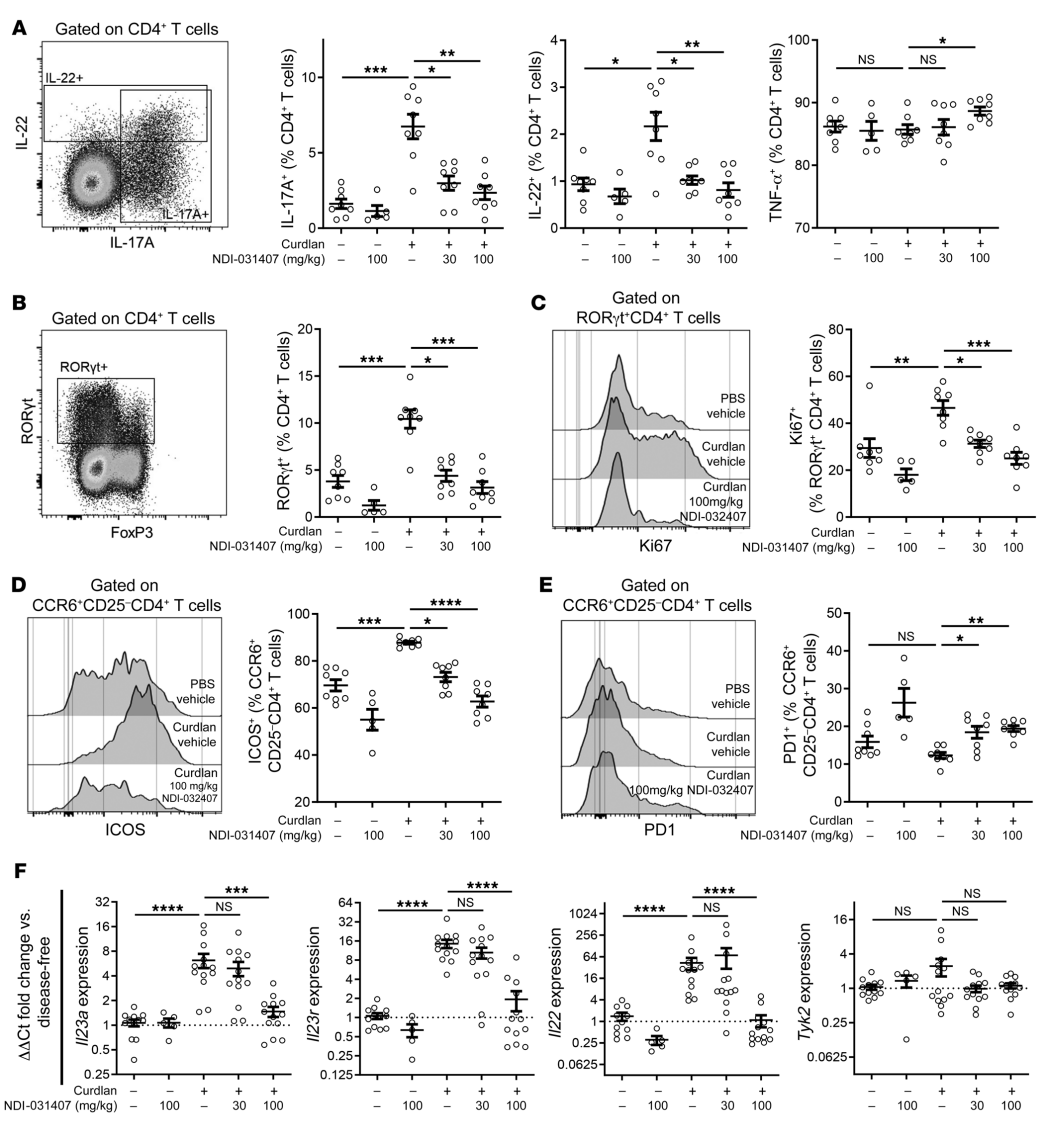
TYK2 inhibition by small molecule normalizes the Th17 expansion in diseased SKG mice. (**A**–**E**) Flow cytometry performed on sciatic lymph nodes (SLNs) at 8 weeks after disease induction (curdlan), with TYK2 inhibitor (NDI-031407) treatment beginning 1 week after disease induction. SLN cells were restimulated with PMA/ionomycin before staining for selected cytokines (**A**). Unstimulated SLN cells were stained for transcription factors (**B**), Ki67 (**C**), and the activation markers ICOS (**D**) and PD1 (**E**). Representative plots in **A** and **B** from diseased, vehicle-treated SKG mouse. (**F**) qPCR performed on mRNA extracted from skinless, whole ankles for selected Th17-associated genes and *Tyk2*. Genes of interest were normalized to *Rpl4*. For all graphs, disease-free mice compared with curdlan/vehicle-treated mice by Mann-Whitney test. Curdlan/NDI-031407–treated mice compared with curdlan/vehicle controls by Kruskal-Wallis test with Dunn’s post hoc test. Each data point represents a single mouse. *P < 0.05, **P < 0.01, ***P < 0.001, ****P < 0.0001.

As production of IL-17A is the operative definition of Th17 cells, we first examined CD4^+^ T cell cytokine expression in PMA/ionomycin-restimulated lymph node samples (gating strategy shown in Supplemental Figure 6A). IL-17A^+^CD4^+^ T cells were indeed increased in frequency in the joint-draining lymph nodes of diseased SKG mice (Figure 3A and Supplemental Figure 5C). CD4^+^ T cells expressing other Th17-related cytokines, such as IL-22 and IL-17F, were more frequent in joint-draining lymph nodes; however, the frequency of the Th1-associated cytokine IFN-γ or the nonspecific cytokine TNF-α was unchanged with SpA induction (Figure 3A and Supplemental Figure 5B). Treatment with NDI-031407 was able to reduce the frequency of CD4^+^ T cells expressing IL-22, IL-17A, and IL-17F to that seen in disease-free mice. Interestingly, no change was seen in the IL-17A production of CD4^+^ T cells from the MLNs (Supplemental Figure 5C).

A recognized caveat with PMA/ionomycin restimulation is that it may not reflect the true phenotype of the cells in vivo. To address this issue, we stained unstimulated lymph node cells for transcription factors (gating shown in Supplemental Figure 6B). The expression pattern of RORγt CD4^+^ T cells, a key transcription factor that regulates Th17 cell function, mirrored that of IL-17A in joint-draining lymphocytes. Specifically, RORγt^+^CD4^+^ T cells were increased in frequency with SpA induction, and were reduced in frequency with NDI-031407 (Figure 3B and Supplemental Figure 5D). In the same panel, we stained for the transcription factor Ki67, a marker of cell proliferation. Proliferation of RORγt^+^CD4^+^ T cells was significantly increased with SpA, and was reduced by treatment with NDI-031407 (Figure 3C and Supplemental Figure 5E).

To further examine Th17 cell activation, we measured a number of cell surface markers of activation. In this panel, we identified Th17 cells as CCR6^+^CD25^–^CD4^+^ T cells (gating shown in Supplemental Figure 6C). The T cell activation marker ICOS, which is highly expressed by Th17 cells (37), was significantly upregulated with disease, an effect that was reversed by NDI-031407 (Figure 3D). Unexpectedly, Th17 cells did express moderate levels of the immune-suppressive molecule PD1. While this marker was unaltered in the presence of disease, NDI-031407 alone was able to cause PD1 upregulation on Th17 cells (Figure 3E).

We performed quantitative PCR (qPCR) to detect Th17-related genes in the ankle and observed that *Rorc* (RORγt) expression was uninterpretable, likely because of high expression in muscle cells (38). Although we were not able to detect *Il17a*, we did see significant upregulation of *Il23a*, *Il23r*, and *Il22* with SpA; however *Tyk2* expression was unchanged (Figure 3F). The increased expression levels of these genes in diseased mice were reduced to disease-free levels with NDI-031407 treatment.

Thus, flow cytometry and qPCR both showed that treatment of SKG mice with the TYK2 inhibitor NDI-031407 is able to suppress SpA-associated Th17 cell responses in draining lymph nodes and arthritic joints.

### NDI-031407 controls type 3 immune cell activity with systemic IL-23 overexpression.

**Figure 4:**
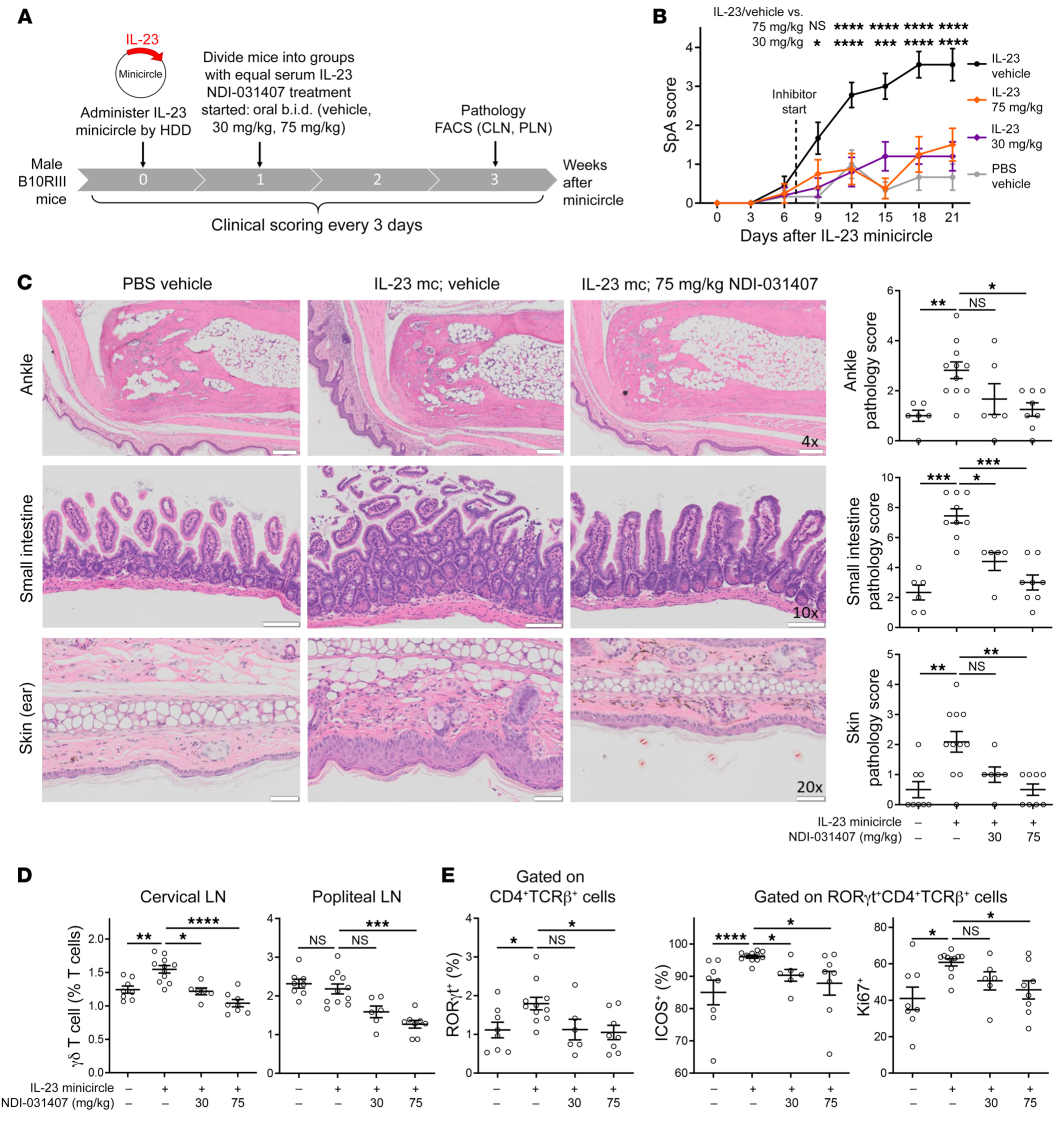
TYK2 inhibition by small molecule suppresses systemic IL-23–induced type 3 immunity in vivo. (**A**) Schematic of experiment. IL-23–expressing minicircle was administered by hydrodynamic delivery (HDD) to male B10RIII mice. At 1 week after minicircle administration, NDI-031407 was administered by gavage twice daily for 2 weeks. Clinical scoring for blepharitis, dermatitis, and arthritis was assessed every 3 days. (**B**) Pooled data for clinical scores (*n* = 5–8 per group). (**C**) Representative images of H&E-stained tissue at 3 weeks after minicircle administration; data pooled from each mouse shown in adjacent graphs. Scale bars: 200 μm for 4×, 100 μm for 10×, 50 μm for 20×. (**D** and **E**) At 3 weeks after minicircle administration, ear-draining (CLNs) and joint-draining (PLNs) lymph nodes were harvested for flow cytometric analysis of T cell subsets by transcription factor expression. (**D**) γδ T cell (TCRγδ^+^TCRβ^–^) frequency in draining lymph nodes. (**E**) Th17 cell frequency and activation status in the PLN. Data in **B** were analyzed by 2-way ANOVA with data paired over time. Average of minicircle/vehicle-treated mice compared with that of minicircle/NDI-031407–treated mice by Dunn’s post hoc test. In all other graphs, disease-free mice compared with minicircle/vehicle-treated mice by Mann-Whitney test and minicircle/NDI-031407 compared with minicircle/vehicle by Kruskal-Wallis test with Dunn’s post hoc test. For all scatter plots, each point represents a single mouse. *P < 0.05, **P < 0.01, ***P < 0.001, ****P < 0.0001.

To further investigate the role of TYK2 in the IL-23 response in vivo, we used the IL-23 minicircle model of SpA (39). Based on these studies, we opted for a short time course of 3 weeks (Figure 4A). In our hands, dermatitis onset was rapid (4–7 days), as was enteritis, reflected by a rapid weight loss and significantly inflamed small intestine evident upon necropsy. Arthritis onset in the paws occurred at 2 weeks; however, arthritis of the tail was conspicuously absent for the duration of our experiment (Supplemental Figure 7). The TYK2-saturating dose of 100 mg/kg NDI-031407 used in the SKG studies was incompatible with the IL-23 minicircle model because of an exacerbation of acute enteritis. This is likely a result of the interference with the protective role of IL-22 on intestinal epithelial cells (40). A subsaturating dose of 75 mg/kg was well tolerated in the IL-23 minicircle model, so was used for experiments.

Treatment groups were randomized so that serum IL-23 levels at 7 days after minicircle were comparable (Supplemental Figure 7). Clinical scoring revealed that twice-daily treatment of NDI-031407 was protective against disease driven by systemic IL-23 overexpression (Figure 4B). H&E staining of tissues at endpoint showed clear inflammation of the small intestine and skin (Figure 4C), consistent with our clinical observations of dermatitis and weight loss. Peripheral arthritis was not as severe in the IL-23 minicircle model as that seen in the SKG model, which might be due to the shorter duration of the experiment. Despite this, IL-23 did cause significant pathology, driven primarily by enthesis-related synovitis and bone marrow edema. Limited bone erosion was seen. NDI-031407 was able to protect against pathological changes to the above tissues, despite the continued presence of IL-23 in the serum at endpoint (not shown).

In the IL-23 minicircle model, enthesitis is reported to be dependent on γδ T cells, rather than Th17 cells (41, 42). We examined the cervical lymph nodes (CLNs) because of the pronounced dermatitis in the ears, and the PLNs because of the peripheral arthritis. Both sets of lymph nodes were visibly enlarged with the IL-23 minicircle administration, reflected by their increase in cellularity (Supplemental Figure 8A). We then examined γδ T cell frequency by FACS (gating strategy shown in Supplemental Figure 8, B and C), and found that IL-23 increased γδ T cell frequency only in the CLNs (Figure 4D). The introduction of NDI-031407 decreased γδ T cell frequency in both the PLNs and the CLNs. A pathogenic role for CD4^+^ T cells in mice treated with IL-23 minicircle has been alluded to through protection mediated by anti-CD4 treatment (43). In support of a role for adaptive immunity in the IL-23 minicircle model, we observed an increased Th17 frequency with IL-23 overexpression (Figure 4E). IL-23–stimulated Th17 cells had an activated phenotype as judged by increased expression of ICOS and Ki67. Consistent with our SKG experiments, NDI-031407 was able to normalize Th17 frequency and reduce activation markers in mice with IL-23–induced SpA.

In summary, while the SKG and IL-23 minicircle models differ in their clinical phenotype and etiology, TYK2 blockade by a small-molecule inhibitor is able to provide a consistent therapeutic effect.

### TYK2 inhibition by NDI-031407 or genetic inactivation protects against dermal γδ T cell activation.

**Figure 5:**
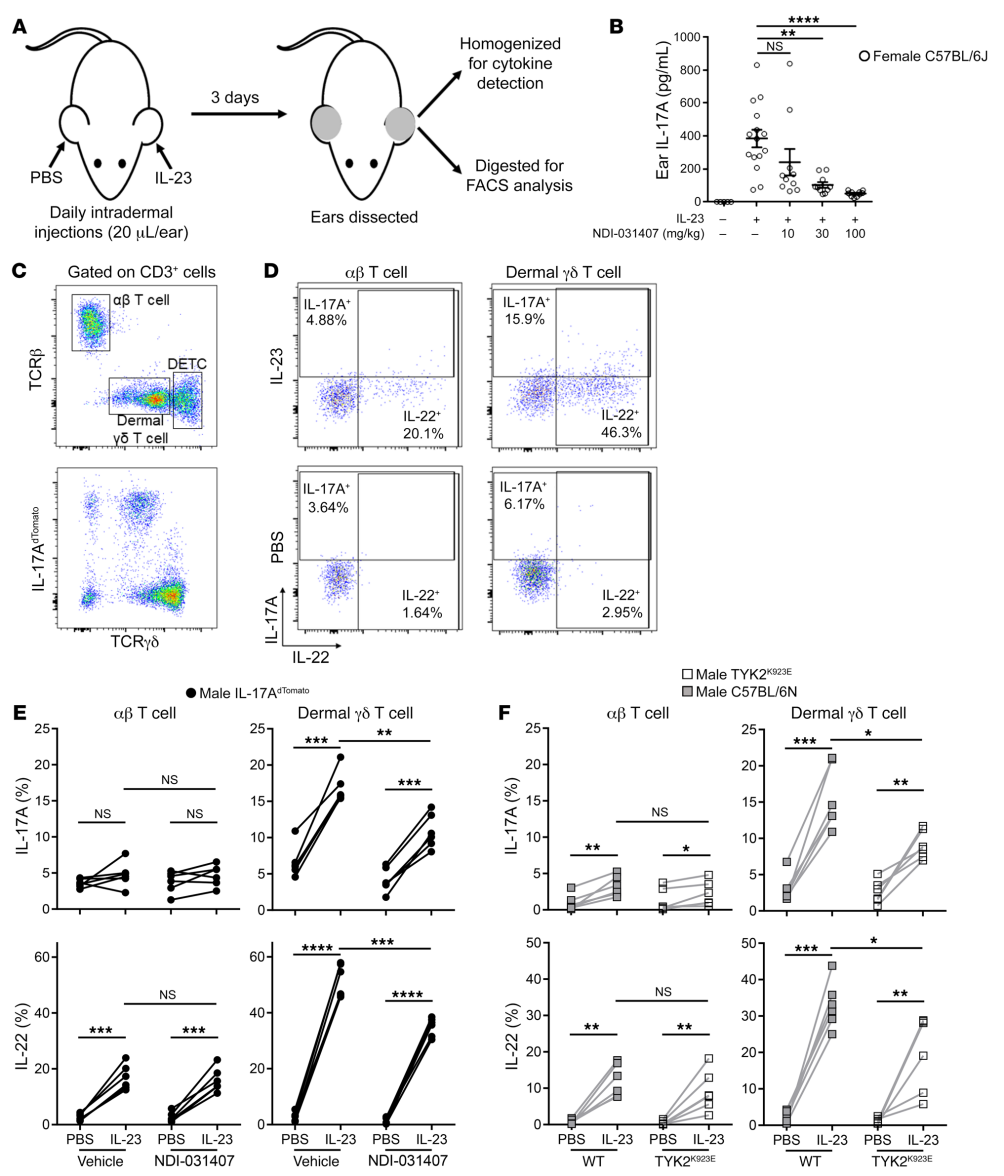
TYK2 inhibition by small molecule or genetic mutation suppresses local IL-23–induced type 3 immunity in vivo. (**A**) Overview of intradermal model of local IL-23 inflammation. IL-23 (400 ng) was administered by intradermal injection into one ear and PBS into the contralateral ear of mice for 3 consecutive days. For experiments involving NDI-031407, the indicated dose or 100 mg/kg was administered by gavage twice daily, starting 1 day before ear injections. (**B**) IL-17A assessed in whole-ear homogenate by Luminex assay. (**C**) Ears from a healthy mouse were enzymatically digested for analysis by flow cytometry. Dermal/epidermal T cell populations were identified in live CD45^+^CD3^+^ cells (top) and cytokines were assessed by staining for IL-17 and IL-22 in *Il17a^Cre^.Rosa26^dTomato^* (IL-17A^dTomato^) reporter mice (bottom). (**D**–**F**) Brefeldin A was administered i.p. 5 hours before mouse sacrifice for intracellular cytokine analysis by flow cytometry. (**D**) Representative plots showing cytokine expression in dermal T cell populations of vehicle-treated mice. (**E**) Pooled data demonstrating in vivo IL-23–induced T cell IL-17A/IL-22 in the presence of NDI-031407. (**F**) In vivo IL-23–induced T cell IL-17A/IL-22 in mice expressing a kinase-inactive TYK2 (TYK2^K923E^). For all graphs, IL-23–treated ear compared with PBS-treated ear by paired *t* test; IL-23–treated ears from vehicle vs. NDI-031407 or WT vs. TYK2^K923E^ compared by unpaired *t* test with Welch’s correction. For graphs in **B**, **E**, and **F**, each point represents data from a single mouse. *P < 0.05, **P < 0.01, ***P < 0.001, ****P < 0.0001.

To address the role of TYK2 in the activation of tissue-resident type 3 immune cells, we turned to a model of local IL-23–induced ear dermatitis. In this model, IL-23 is injected intradermally to induce rapid, local γδ T cell activation (44), with the contralateral ear used as a sham injection control (Figure 5A). We first measured cytokines in whole ear tissue. Here we detected robust levels of IL-17A in homogenized ears after 3 days of intradermal IL-23 administration (Figure 5B). Oral treatment with NDI-031407 was able to block IL-17A expression in the ear (Figure 5B).

We next performed immune phenotyping of T cell infiltrates in the ears. There are 3 major T cell populations in murine skin (44): dermal αβ T cells, dermal γδ T cells, and dendritic epidermal γδ T cells (DETCs) (Figure 5C). Using IL-17A fate-mapping mice, we showed that all dermal γδ T cells and some dermal αβ T cells had produced IL-17A (IL-17A^dTomato+^) at some point during their lifespan (Figure 5C). IL-17A^dTomato^ was poorly expressed in DETCs from both uninflamed and IL-23–treated ears, so this cell population was not examined in further experiments.

As reported (44), we were able to directly detect intracellular cytokines in dermal T cells without the need for ex vivo restimulation by treating the mice with brefeldin A before sacrifice. Representative plots in Figure 5D illustrate that T cells from the PBS-treated ears express very little IL-17A/IL-22, while IL-23 is able to drive robust expression of IL-22, especially in dermal γδ T cells. IL-17A was also induced, albeit at lower frequencies than IL-22. Cytokines were not detectable in the ear-draining CLNs (Supplemental Figure 9), supporting the notion that this truly is a model of local inflammation.

As with our other in vivo models, TYK2 inhibition with NDI-031407 reduced the activation of IL-23–activated type 3 immune cells. More specifically, NDI-031407 lowered the frequency of IL-23–induced IL-17A/IL-22 in dermal γδ, but not αβ, T cells (Figure 5E).

This model can be performed on the C57BL/6 background, unlike the SKG and IL-23 minicircle models, which allowed us to dissect the role of TYK2 using genetically modified mice. We performed the same experiment in mice with enzymatically inactive TYK2 through targeted mutation of the kinase domain (TYK2^K923E^) (45). Genetic inactivation of TYK2 in mice yielded results almost indistinguishable from those seen with small-molecule inhibition (Figure 5F), providing strong support that the in vivo effects of NDI-031407 are TYK2-specific. Importantly, treatment of TYK2^K923E^ mice with NDI-031407 did not result in appreciable suppression of IL-17 or IL-22 (Supplemental Figure 9B), supporting its on-target inhibition.

### TYK2 is essential for IL-23–induced STAT3 phosphorylation in murine γδ T cells.

**Figure 6:**
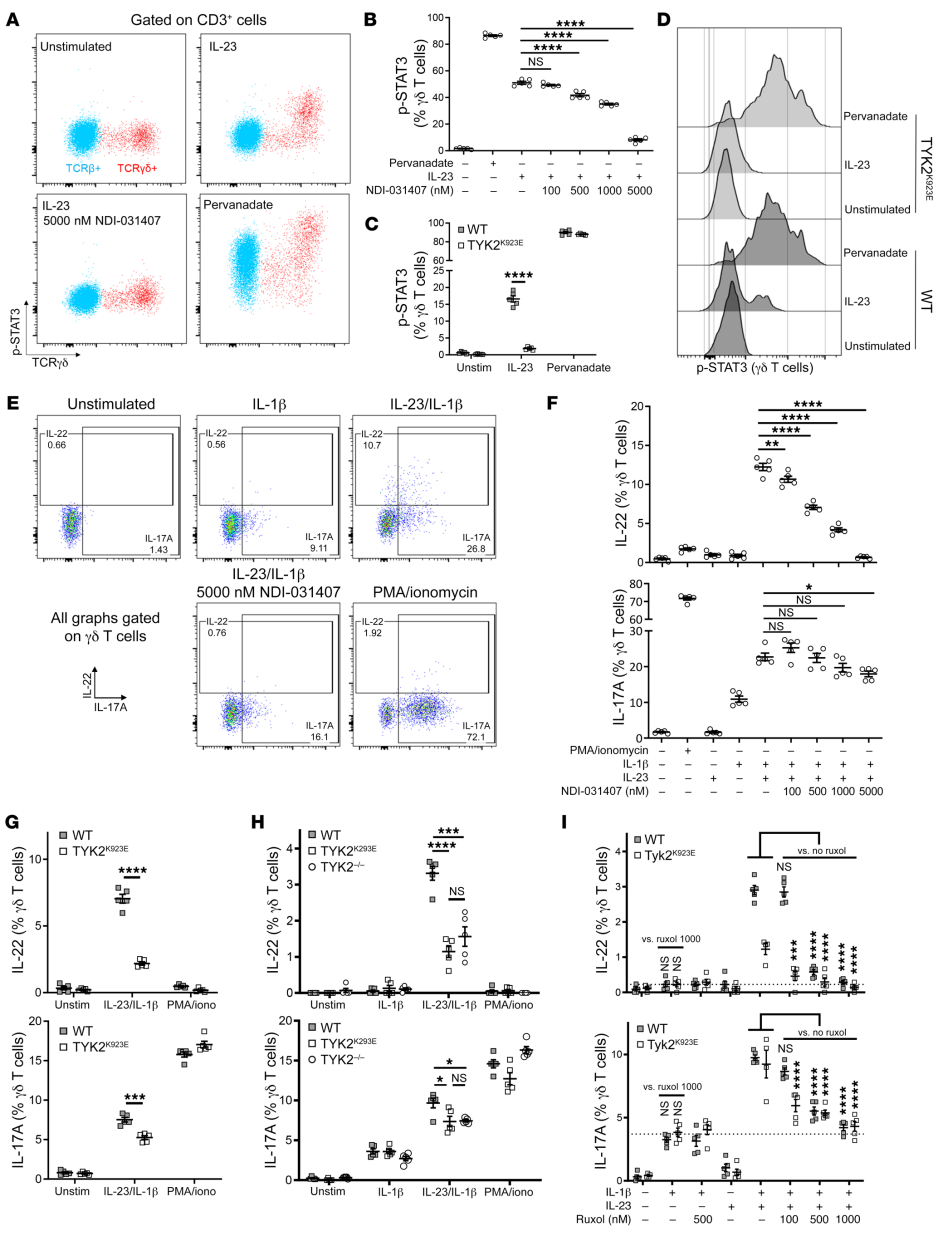
TYK2 inhibition by small molecule and genetic mutation suppresses IL-23–induced activation of murine T cells in vitro. Lymph node cells were stimulated in vitro, and γδ T cell activation was assessed by flow cytometry. (**A** and **B**) Lymphocytes were preincubated with NDI-031407 for 30 minutes before stimulation for 15 minutes with 400 ng/mL IL-23. (**A**) Representative plots showing p-STAT3 staining under IL-23 or pervanadate stimulation in αβ and γδ T cells. (**B**) Pooled data of NDI-031407–treated, IL-23–stimulated γδ T cells. (**C** and **D**) Representative plots and pooled data of p-STAT3 in γδ T cells under IL-23 stimulation in TYK2 kinase-dead mice (TYK2^K923E^). (**E**–**H**) Lymphocytes were stimulated with 10 ng/mL IL-1β and/or 20 ng/mL IL-23 with brefeldin A for 4.5 hours before detection of IL-17A/IL-22 in γδ T cells by flow cytometry. Where applicable, lymphocytes were treated with NDI-031407 for 30 minutes before stimulation. (**E**) Representative plots showing cytokine staining in γδ T cells. (**F**) Pooled data of IL-17A^+^ and IL-22^+^ γδ T cells treated with NDI-031407. (**G** and **H**) IL-23/IL-1β stimulation of TYK2^K923E^ (**G**) and TYK2^–/–^ (**H**) lymphocytes. (**I**) IL-23/IL-1β stimulation of TYK2^K923E^ lymphocytes with pan-JAK inhibitor ruxolitinib. Data are mean ± SEM whereby each data point is a separate well. All data are from a single experiment representative of 2–3 independent experiments. **D** and **G**, *t* test with Welch’s correction; **B** and **F**, 1-way ANOVA with Dunnett’s post hoc test compared with cytokine-stimulated/vehicle control; **H**, 1-way ANOVA with Dunnett’s post hoc test. In **I**, IL-1β only was compared with IL-1β/IL-23/1000 nM ruxolitinib for each genotype by *t* test with Welch’s correction, and 2-way ANOVA was used to compare all IL-1β/IL-23–stimulated cells and Dunnett’s multiple-comparisons test to compare vehicle- vs. ruxolitinib-treated samples within each genotype. *P < 0.05, **P < 0.01, ***P < 0.001, ****P < 0.0001.

Given the rapid response of γδ T cells to IL-23 in vivo, we decided to use these cells as model type 3 immune cells in vitro to further dissect the molecular function of TYK2 in IL-23 signaling. The dosage of NDI-031407 required for murine immune cells is naturally higher than that in the aforementioned human assays, as NDI-031407 was designed against human TYK2 and is thus less efficacious against its murine homolog. We first tested intracellular signaling of IL-23 in freshly isolated lymph node cells without concurrent cytokine or TCR stimulation. Here, we observed clear STAT3 phosphorylation after 15 minutes of IL-23 stimulation in γδ, but not αβ, T cells (Figure 6A). This activity was strain-dependent, with BALB/c mice possessing a more robust response to IL-23 than C57BL/6. Accordingly, BALB/c lymphocytes were used for in vitro assays with NDI-031407. As can be seen in Figure 6B, TYK2 inhibition by NDI-031407 was able to interfere with IL-23–induced STAT3 phosphorylation in a dose-dependent fashion. We repeated this phosphoflow assay with cells from TYK2^K923E^ mice or WT (C57BL/6N) controls. p-STAT3 was strikingly absent after IL-23 stimulation in cells from TYK2^K923E^ mice (Figure 6, C and D), supporting the on-target effect of NDI-031407.

### IL-23–induced IL-17A is only partially dependent on the TYK2/p-STAT3 pathway.

In order to assess the effect of TYK2 on cytokines downstream of the IL-23R, we stimulated the lymph node extracts for 4.5 hours. As reported (44), γδ T cells produce IL-17A and IL-22 with the combination of IL-1β and IL-23 (Figure 6E). IL-1β signaling is NF-κB–dependent and STAT-independent (46). Interestingly, IL-17A was slightly induced by IL-1β alone, whereas IL-22 was not. The duality of the regulation of these cytokines is also highlighted by their induction with PMA/ionomycin: IL-22 appears to be insensitive to calcium signaling, unlike IL-17A. Further, even though IL-23 was able to cause STAT3 phosphorylation, IL-23 alone was unable to induce the expression of effector cytokines, as it required costimulation with IL-1β. IL-23/IL-1β–induced IL-22 was completely inhibited by NDI-031407, whereas IL-17A was only partially inhibited even at the highest dose (Figure 6F). The results were remarkably similar in TYK2^K923E^ γδ T cells (Figure 6G). We therefore conclude that IL-23 induces IL-17A largely independently of TYK2/p-STAT3, yet IL-22 is strictly TYK2/p-STAT3–dependent.

The differential regulation of IL-17A and IL-22, both type 3 immune cell effector cytokines, challenges the current dogma of their mutual coexpression. Aside from their kinase activity, JAKs can also influence intracellular signaling through scaffolding functions (47). To test the hypothesis that TYK2 may be acting as a scaffold protein in the induction of IL-17A by IL-23, we conducted our in vitro cytokine assay in TYK2^–/–^ lymphocytes. The complete absence of TYK2 did not lower IL-17A (or IL-22) production in γδ T cells compared with the presence of noncatalytic TYK2^K923E^ (Figure 6H). We then considered the hypothesis that IL-23 may synergize with IL-1β to activate IL-17A through JAK2, which is also associated with the IL-23R (48). Ruxolitinib, a pan-JAK inhibitor, was able to reduce IL-1β/IL-23–induced IL-17A to the levels seen with IL-1β stimulation only (Figure 6I). This same assay provides evidence that JAK2 acts in synergy with TYK2 in the induction of IL-22, likely through STAT3 phosphorylation as suggested by strong p-STAT3 inhibition by ruxolitinib shown in our PBMC assays (Figure 1G).

In summary, our in vitro assays have shown that IL-23R complex activation engages both JAK2 and TYK2. While the induction of the effector cytokine IL-22 occurs via JAK2-TYK2/p-STAT3, we show that IL-23–activated JAK2 promotes IL-17A production largely independently of TYK2/p-STAT3 (Supplemental Figure 10).

### TYK2 SNPs associated with AS do not affect TYK2 expression.

In order to examine the case for using TYK2 inhibitors therapeutically in SpA, we examined the effect of GWAS-linked *TYK2* SNPs on *TYK2* expression and function. The first *TYK2* SNP to be associated with SpA (AS) was rs35164067 (24), located 34 kb upstream of the *TYK2* gene. As seminal studies in the field have shown that many autoimmune-associated SNPs function as super-enhancers (49), we reasoned that this SNP may modulate *TYK2* expression. To test this hypothesis, we assembled a cohort of AS patients from our clinic, with rheumatoid arthritis patients and healthy individuals as controls (RA controls and HCs, respectively) (Supplemental Table 1). The HCs were age- and sex-matched to the AS patients; however, the RA controls were older and predominantly female because of the different demographics of the disease.

**Figure 7:**
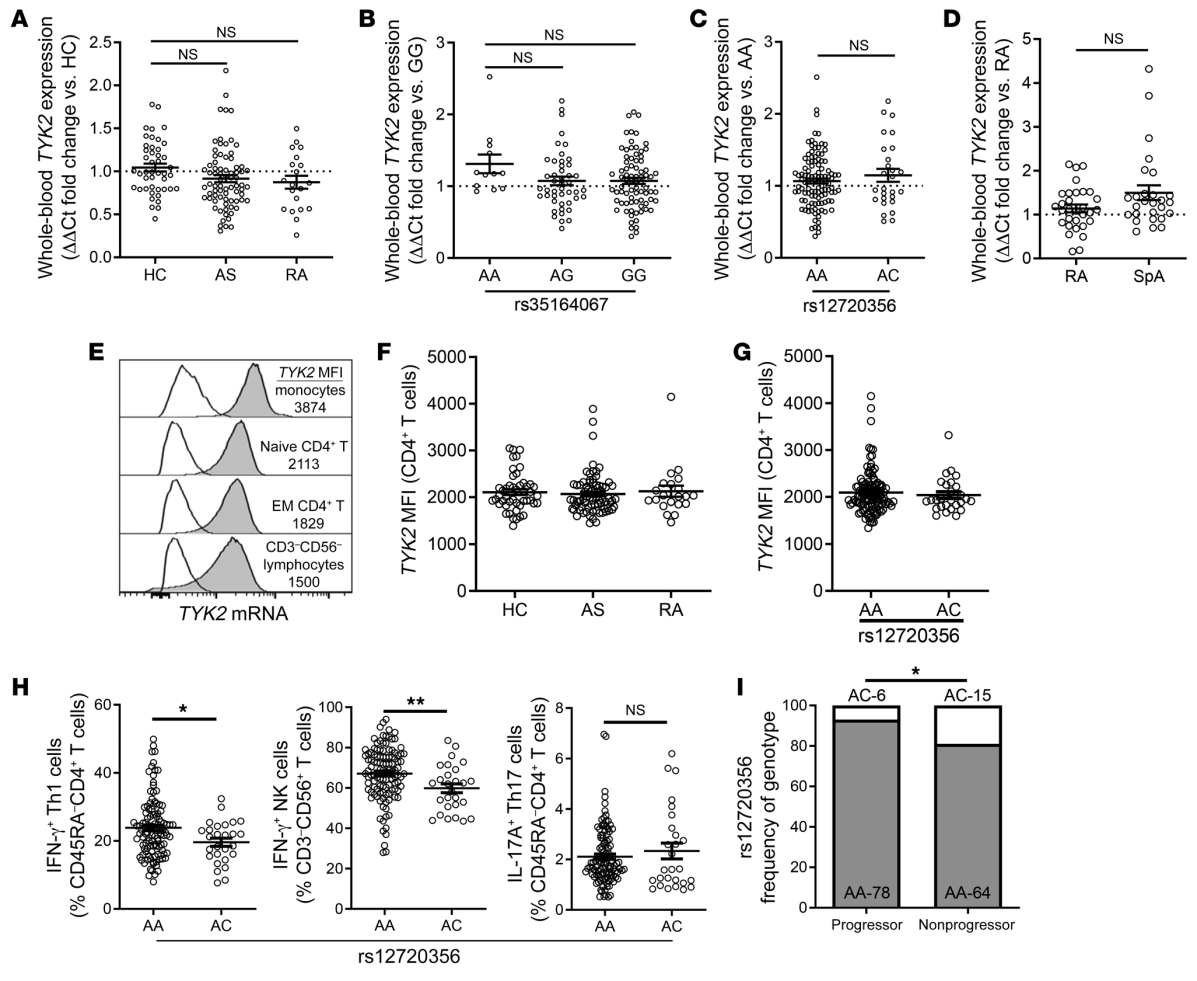
AS-associated SNPs at the *TYK2* locus do not alter *TYK2* expression, but correlate with altered Th1 frequency and AS disease progression. qPCR was used to assess whole-blood *TYK2* expression in a cohort of 47 healthy controls (HC), 76 ankylosing spondylitis patients (AS), and 21 rheumatoid arthritis patients (RA) by patient type (**A**) and rs35164067 (**B**) and rs12720356 (**C**) genotypes. (**D**) *TYK2* expression in peripheral joint synovial biopsies measured by qPCR. PrimeFlow was used to detect *TYK2* mRNA by flow cytometry. (**E**) Representative histograms showing *TYK2* mRNA expression in selected cell populations. White histograms represent FMO controls, gray histograms represent *TYK2*-stained cells. Values under cell populations are the respective MFIs of *TYK2*. (**F**) *TYK2* MFI in CD4^+^ T cells by patient group. (**G**) AS/RA/HC subjects were pooled to assess *TYK2* expression by rs12720356 genotype. (**H**) PBMCs from the same cohort were stimulated with PMA/ionomycin for IL-17A and IFN-γ detection by flow cytometry. AS, RA, and HC pooled and data stratified by rs12720356. (**I**) Frequency chart of rs12720356 genotype assessed in a separate cohort of AS patients with progressing (*n* = 84) or nonprogressing (*n* = 79) disease based on mSASSS scores. qPCR analysis in **A**–**C** was normalized to *HPRT* expression and to *GAPDH* in **D**. **A** and **B**, 1-way ANOVA with Tukey post hoc test; **C**, **D**, and **H**, Mann-Whitney test; **I**, Fisher’s exact test. *P < 0.05, **P < 0.01.

We first assessed *TYK2* mRNA expression in whole blood by qPCR. Here we found no evidence of differential *TYK2* expression between AS patients and controls (Figure 7A). We then pooled all subjects in the cohort and reanalyzed *TYK2* expression by rs35164067 (Figure 7B), and found no evidence that this SNP altered *TYK2* expression. Recent meta-analyses found a collection of exonic *TYK2* risk SNPs to be associated with AS (22, 23). Owing to the low minor allele frequency of these SNPs (Supplemental Figure 1A), we were only able to perform post hoc analysis on rs12720356 (I684S). This *TYK2* SNP also had no effect on *TYK2* expression (Figure 7C).

It is possible that the expression profile of *TYK2* in the peripheral blood does not represent its expression in the disease-relevant target organs or tissues. To address the possibility that *TYK2* expression may be altered in inflamed joint tissue, we measured *TYK2* expression by qPCR in synovial biopsy samples from early SpA patients (Supplemental Table 2). We saw no difference in the expression of *TYK2* between SpA patients and RA controls (Figure 7D). The lack of differential *TYK2* expression in the joints of SpA patients mirrors our observations of static *TYK2* expression in the joints of arthritic SKG mice (Figure 3F).

A final possibility is that AS-associated *TYK2* SNPs may be acting in a cell-specific manner, and thus their effects are not detectable in samples of mixed cell origin. No validated antibodies are available for flow cytometric evaluation of TYK2. To overcome this hurdle, we used a custom PrimeFlow kit (eBiosciences), which allows for mRNA detection by conventional flow cytometry. The cohort of AS patients and controls used for PrimeFlow was the same as that used for whole-blood qPCR (Figure 7A). We designed a multicolor panel to assess *TYK2* expression across a range of immune cell types in PBMCs (gating shown in Supplemental Figure 11A). *TYK2* mRNA was clearly detectable across cell types tested, as illustrated in representative histograms in Figure 7E. We found no differential expression of *TYK2* between AS patients and controls in any cell type tested, as demonstrated in CD4^+^ T cells (Figure 7F). We further pooled all subjects to analyze cellular *TYK2* expression stratified by rs12720356 and rs35164067 (Figure 7G and Supplemental Figure 11B) and found no differential expression of *TYK2* by AS-associated SNPs in all cell types assessed, in line with our findings in whole-blood RNA.

Despite not seeing any difference in *TYK2* expression with disease status or SNP of interest, we did observe differences in expression by cell type. Monocytes had the highest level of *TYK2* expression, with further variation seen among major lymphocyte populations (Supplemental Figure 11C). In addition, *TYK2* expression appeared higher in naive T cells than in their mature counterparts (Supplemental Figure 11D). To confirm this finding, we sorted key cell populations by FACS from 5 healthy individuals and measured *TYK2* expression by qPCR (Supplemental Figure 12).

In sum, extensive gene expression data in whole blood, at the cellular level in PBMCs and in synovial tissue, indicated that *TYK2* expression is not altered with SpA, nor is it affected by AS-associated risk SNPs studied here.

### AS-associated TYK2 SNPs correlate with altered immune cell and SpA phenotype.

As the recently identified *TYK2* SNPs associated with AS impart or are linked to TYK2 protein-coding changes, we addressed how these SNPs might modify T cell phenotype and disease severity. One of the SNPs associated with AS, rs34536443 (P1104A), results in LoF and is protective against a broad range of autoimmune diseases (23). It has also been demonstrated that rs34536443 reduces signaling through TYK2-associated cytokine receptor complexes such as IL-12R and IL-23R (23). While our cohort size made it difficult to assess the role of rs34536443, we were able to assess the effect of rs12720356 (I684S), also a LoF variant that is linked to a protection against multiple immune diseases (23, 25). In contrast to the protective rs34536443 SNP in *TYK2*, rs12720356 is associated with a paradoxical increased risk for the onset of AS and IBD; however, its effect on disease severity is not known.

We tested the effect of rs12720356 on T cell polarization through PMA/ionomycin restimulation (gating strategy shown in Supplemental Figure 13A). As we and others have previously reported (50, 51), AS patients had elevated IL-17A^+^CD4^+^ (Th17) and reduced IFN-γ^+^CD4^+^ (Th1) cell frequencies (Supplemental Figure 13, B and C). The intergenic SNP rs35164067 had no effect on T cell phenotype (Supplemental Figure 13D); however, subjects heterozygous for rs12720356 had reduced frequencies of Th1 cells and IFN-γ^+^ NK cells (Figure 7H). This result is in line with the dependence of these cell types on IL-12 signaling and the reduced Th1 differentiation in TYK2-deficient mice (21). We did not see an effect of rs12720356 on Th17 cells. This is consistent with the role of IL-23 in sustaining Th17 phenotype, but not differentiation (52), and the lack of effect of TYK2 deficiency on Th17 cell frequency in both humans and mice (17, 21). It is possible that rs12720356 is altering Th17 phenotype in peripheral tissue; however, we could not test this hypothesis owing to the lack of access to human arthritic joint tissue that would be required to examine sufficient numbers of rs12720356 carriers.

Given the correlation of rs12720356 with immune cell polarization, we speculated that carriage of rs12720356 might impact the severity of AS. To test this, we assembled a cohort of patients to compare those with progressive disease, measured by a high rate of vertebral fusion (mSASSS > 1 U/yr), with those with low rates of vertebral fusion (Supplemental Table 3). The frequency of the rs12720356 minor (LoF) allele was significantly higher in the AS nonprogressors, suggesting it has a protective effect against AS disease progression (Figure 7I). Since carriage of rs12720356 protects against disease progression, likely by hampering T cell activation, TYK2 appears to be a promising therapeutic target in AS (23, 29).

## Discussion

The development of JAKinibs for inflammatory diseases is an area of active research, with tofacitinib approved for RA and PsA, and many other JAKinibs in phase III trials (15, 53). While there are multiple inhibitors under development for JAK1–3 in autoimmune diseases, there are few TYK2-selective inhibitors, with only one other agent claiming to be TYK2-specific, BMS-986165 (53). BMS-986165 was recently reported as an effective therapeutic for psoriasis in phase II trials (54), and has been shown to inhibit the pseudokinase domain of TYK2 to provide a therapeutic effect against type I IFN– and IL-12–dependent autoimmune disease models in vivo (55). It has further been reported that nonspecific TYK2 inhibitors are effective at blocking IL-23–mediated inflammatory skin disease (56), in line with in vivo reports in TYK2-deficient mice (20). In this paper, we provide in vitro and in vivo evidence that a novel TYK2-specific inhibitor mediates its protective effect through inhibition of type 3 immune cells, namely Th17 and γδ T cells.

There are no animal models that faithfully reproduce the clinical phenotype of SpA. We used two IL-23–dependent models, the SKG and IL-23 minicircle, that symptomatically resemble mixed AS and PsA (31, 32, 39). We show that TYK2 inhibition in these SpA models had a protective effect associated with inhibition of type 3 immunity. It is of importance that these models have considerably different underlying immune mechanisms, the SKG model being Th17-dependent and chronic and the minicircle model being γδ T cell–dependent and acute (36, 39). Clinically, both cell types are relevant to SpA: Th17 cells have been implicated in AS and PsA in multiple studies (7), while γδ T cells are less studied; they have been found to be more IL-17–skewed in AS and can be readily found in human ligaments (57, 58). These results should be interpreted with the recognition, however, that human γδ T cells respond poorly to IL-23 compared with their murine counterparts (59), and tend to make up a lower proportion of tissue-resident cells in humans (60). This latter observation might be an artifact of housing mice under clean conditions: γδ T cells are the first wave of neonatal tissue-resident T cells (60); however, αβ T cells dominate upon microbial exposure (61).

In vitro, our data confirm previous reports that IL-23 does not signal exclusively through STAT3 (62). With the new tools available, we have extended these observations to show, for the first time to our knowledge, that IL-17A and IL-22 are regulated by distinct IL-23 intracellular signaling pathways. While IL-22 is highly dependent on JAK2/TYK2/STAT3 signaling, TYK2 and subsequent STAT3 phosphorylation only partially contribute to IL-23 promotion of IL-17A, whereas JAK2 is essential. How JAK2 is mediating IL-17A induction is not clear, but it is likely to be through modulation of NF-κB (63, 64), which provides a direct mechanistic link for IL-23’s ability to enhance IL-1β–induced IL-17A. In vivo, TYK2 is clearly involved in regulating IL-22 expression, but also likely modulates IL-22 function given the association of TYK2 with IL-22R (40). While we did not explore this pathway, it is of great interest in the context of SpA given the tentative role of IL-22 in new bone formation (65): IL-22 overexpression by minicircle, but not IL-17A overexpression, is able to reproduce the arthritic phenotype of IL-23 overexpression (39). Further, blocking IL-22 is antiarthritic in both the minicircle and SKG models of SpA (31, 39).

Recent trials with anti–IL-23 agents have raised new questions about the pathogenesis of AS. Anti–IL-23 is beneficial for PsA (66) and the SpA-related diseases psoriasis and IBD (31, 39). An open-labeled trial with an IL-12/IL-23 dual blocker initially suggested efficacy in AS (67), but a subsequent randomized control trial failed to achieve the primary target outcome (68). A recent trial with an IL-23–specific antibody in AS similarly failed to achieve the primary endpoints (69). Despite this, anti–IL-17A therapy is effective and approved for AS. While this work does not explain why blocking IL-23 may be ineffective in AS, it does shed light on the dissociation between IL-23 and IL-17A. As has previously been reported, we have demonstrated that IL-23 alone cannot drive an IL-17A response, despite inducing clear phosphorylation of STAT3. Costimulation, especially with IL-1β, is essential for IL-23 to induce effector cytokines. Animal models provide differing results with regard to the role of IL-23 in SpA pathogenesis. The SKG model shows both therapeutic and prophylactic benefit of blocking IL-23 (32), while a rat model of SpA shows a prophylactic but not a therapeutic effect of blocking IL-23 (70). Notably, blocking IL-23 in this latter model had no effect on the overexpression of IL-17A or IL-22. Such knowledge challenges the conventional wisdom that IL-23 directly translates to IL-17A, an assumption that has been challenged by the aforementioned clinical trial results.

GWAS data have revolutionized our understanding of genetic factors that contribute to the risk of autoimmune diseases. By clustering of disease-associated genes based on their known functions, certain biological pathways have been discovered to play a role in disease pathogenesis. In SpA, there are numerous GWAS-identified genes that are known to modulate the function of type 3 immune cells, *TYK2* being one example through its known function in IL-23R signaling. How risk SNPs, or associated genes, act to modulate disease pathogenesis requires detailed experimentation. Such studies are hampered by many genes being mislabeled as risk factors based on their proximity to disease-associated SNPs. Further, most disease-associated SNPs are not the primary risk alleles, but are rather linked to those that are (71). While coding variant SNPs may have predictable effects on protein function, intergenic SNPs can affect gene expression from afar, and may do so in a cell- and stimulation-dependent way (49).

In our study, we found no association between the first *TYK2* SNP to be linked to AS, rs35164067, and *TYK2* expression or function. Thus it might be that this SNP, or a neighboring SNP in linkage disequilibrium, mediates its effect on AS risk through other genes in the locus such as *CDC37* or *PDE4A*. Indeed, *CDC37* broadly regulates non-JAK kinases (72), and *PDE4* isoforms modulate immune signaling and are actively being targeted for autoimmune disease (73).

Our results in AS patients support the notion that other *TYK2* SNPs associated with AS (and with PsA) likely exert their effects by altering TYK2 function. It has been reported that *TYK2* LoF exonic SNPs are the primary variants associated with autoimmune disease in the *TYK2* locus (23, 27). Consistent with this, we find the LoF SNP rs12720356 to be associated with reduced frequency of IL-12–dependent cells (Th1 and NK) and to associate with protection against disease progression. We acknowledge that this LoF SNP paradoxically increases the risk of developing AS, despite protecting against a range of other autoimmune diseases (22, 23). As rs12720356 also increases the risk of developing IBD (23), it is possible that a LoF may promote inflammation in mucosal tissues where IL-17 is protective, while being protective at the joint where IL-17 is pathogenic. The protective effect of TYK2 on the gut may reflect the detrimental effect we observed of high-dose NDI-031407 on mice with acute enteritis induced by IL-23 minicircle. The risk/protection mediated through LoF may also result from the location of the mutation (e.g., pseudokinase vs. kinase domain), which will require further investigation. It is worth noting that functional fine-mapping of the AS-associated haplotype that includes rs12720356 has found it is also linked to regulatory regions of additional nearby immune-related genes (such as *ICAM1*), which may also impact the immunophenotype observed in functional studies (23). We did not study the effects of other AS-associated risk alleles that are also LoF since the minor allele frequency would necessitate very large sample sizes. The clustering of AS-associated LoF SNPs in *TYK2* suggests that many variants may mediate similar disease-modifying effects through reducing the function of TYK2. These experiments of nature provide strong evidence that pharmacological inhibition of TYK2 will provide a therapeutic effect in AS.

The study presented here has defined limitations as discussed: We were not able to comprehensively study rare *TYK2* variants in our patient studies. TYK2 may play a protective role in the gut, which should be carefully considered in clinical trials, especially in patients with active enteritis; however, the recent TYK2 inhibitor trial in psoriasis did not report an increased risk of gut-related adverse events in comparison with placebo (54). Finally, the unmet need for therapeutics in AS necessitates inhibition of new bone formation (ankylosis), which is assumed to follow inflammation and bone erosion (74). As no animal models of SpA involve coincident inflammatory bone erosion and new bone formation, we must extrapolate from our results demonstrating protection against bone erosion.

In conclusion, we provide evidence that TYK2 plays an immunomodulatory and pathogenic role in AS. Targeting of TYK2 by small molecule is effective at halting inflammation and erosive changes in animal models of SpA, which suggests it may be an effective disease-modifying therapeutic for AS. This is important as there is an urgent unmet need for such therapies for AS patients, with current therapeutics approved for symptomatic improvement but having fallen short of predictably preventing spinal ankylosis.

## Methods

Supplemental methods are available online with this article.

### Study approval.

To participate, all Canadian patients and controls completed a consent form approved by the research ethics board of the University Health Network (UHN), Toronto, which included informed consent prior to inclusion in the study. All Dutch patients provided written informed consent before enrollment in the study as approved by the Ethics Committee of the Amsterdam Medical Center/University of Amsterdam.

Canadian experiments using animals were approved by the Animal Resource Centre of the UHN, Toronto (protocols 4541, 5499, and 5760), under the guidance of the Canadian Council on Animal Care. Austrian animal breeding was approved by the institutional ethics and animal welfare committee (University of Veterinary Medicine, Vienna) and the national authority (BMWF, Federal Ministry of Education, Science and Research, Vienna, Austria) according to sections 26ff. of the Animal Experiments Act, Tierversuchsgesetz (TVG) 2012 (BMWF 68.205/0068-IWF/3b/2015). The study performed at the University of Veterinary Medicine, Vienna, did not involve animal experiments as defined in the TVG and did not require ethical approval according to the local and national guidelines.

## Author contributions

EG, DH, YB, MM, BS, WM, and RDI designed research studies. EG, DH, ML, ZQ, MZ, YY, AS, YB, and NY conducted experiments. EG, DH, NY, WW, CM, MM, BS, WM, and RDI analyzed data. EG, DH, WW, CM, MM, BS, WM, and RDI wrote the manuscript. EG initiated the project in the laboratory of RDI. DH provided vital experiments in the laboratory of MM. The coauthorship order reflects this.

## Supplementary Material

Supplemental data

## Version 1 03/09/2020

Electronic publication

## Version 2 03/10/2020

Corrections made to figures

## Version 3 04/01/2020

Print issue publication
