# Supplementary material for: TYK2 inhibition reduces type 3 immunity and modifies disease progression in murine spondyloarthritis
Source: J Clin Invest. 2020 Mar 9;130(4):1863–78. doi: 10.1172/JCI126567 (PMC7108927; doi:10.1172/JCI126567)
Supplement: Supplemental data [file jci-130-126567-s308.pdf]

Supplementary Materials and Methods

Patient cohorts and sample isolation

AS and RA patients were recruited from the rheumatology clinic at Toronto Western Hospital, Canada. All AS patients fulfilled the modified New York criteria (1), and RA patients fulfilled the ACR criteria (2). HC were recruited from hospital staff and volunteers with no clinical autoimmune disease or recent infections. For synovial biopsy samples, SpA patients and RA controls were recruited from the Academic Medical Centre (AMC) rheumatology clinic, Amsterdam, Netherlands. All SpA patients fulfilled the ASAS criteria for axial and/or peripheral SpA (3). The clinical characteristics of subjects are provided in Supplementary Tables 1-3.

For PBMC isolation, venous blood was collected in heparinized tubes prior to cell isolation with Ficoll-Paque plus (GE Healthcare). Cells were cryopreserved with 55% FCS and 10% DMSO in RPMI and stored in liquid nitrogen. Thawed PBMC were rested overnight in complete media (RPMI 1640 with L-glut + 10% HI-FCS + penicillin/streptomycin + 50 µM mercaptoethanol) before use.

For whole blood DNA isolation, venous blood was collected in EDTA-treated tubes prior to DNA isolation with a QIAamp blood DNA isolation kit following the manufacturer's protocols (Qiagen). DNA was stored at 4°C.

For whole blood RNA isolation, venous blood was collected in Tempus tubes, and RNA isolation with Spin Isolation Kits (Applied Biosystems). RNA was stored at -80°C.

Synovial tissue biopsies were obtained from clinically inflamed knee or ankle joints and processed as described previously (4). 6-8 biopsies were pooled and used for RNA isolation. Total RNA was extracted from synovial biopsies by homogenization of biopsies in STAT60 (Tel-Test) according to manufacturer's instruction, treated with DNAse, and purified using RNeasy columns (Qiagen).

Murine disease models, treatment and clinical scoring

SKG and BALB/cAJcl mice were obtained from CLEA Japan and were bred in homozygous harems. Mice were cohoused from different breeding cages upon weaning and only female SKG mice were used for arthritis experiments owing to higher disease severity and penetrance vs. male mice (5, 6). Male BALB/cAJcl mice were used for ear dermatitis and γδ in vitro assays. Male B10.RIII mice were obtained from Jackson Laboratories for minicircle experiments. TYK2^{K923E} mice were obtained from M. Müller (Vienna) (7), and bred in homozygous harems for ear dermatitis experiments. Male C57BL/6NCrl were obtained from Charles River as controls. *Il17a*^{cre} mice (Il17atm1.1(icre)Stck/J) and *ROSA26*^{dTomato} (B6.Cg-Gt(ROSA)26Sortm14(CAG-tdTomato)Hze/J) were obtained from Jackson and male F1 offspring (IL-17$^{cre.dTomato}$) were used for ear dermatitis experiments.

For in vitro experiments, C57BL/6N (Janvier Labs), Tyk2-/- (8) and Tyk2^{K923E} (7) mice (on C57BL/6N background), were bred at the University of Veterinary Medicine (Vetmed), Vienna, under specific

pathogen-free conditions according to Federation of European Laboratory Animal Science Associations (FELASA) guidelines. All animals used were between 8-12 weeks of age.

For in vivo experiments involving NDI-031407 (Nimbus Therapeutics), compound was prepared fresh weekly in autoclave-sterilized vehicle (5 g/L 4000cps methylcellulose [Sigma], 10 g/L NaCl, pH 5 in deionized water). Up to 100 mg/kg NDI-031407 was administered oral BID by gavage. Pharmacokinetic experiments performed by Nimbus revealed this dose to provide adequate serum NDI-031407 saturation over a 12 hour period (Supplementary Figure 14). Tofacinib (MedChemExpress) was similarly prepared and administered.

Disease was induced in SKG mice by IP injection of 3 mg curdlan (C7821, Sigma) in PBS. Mice were weighed and scored weekly for clinical signs of SpA. A composite SpA score was created based on presence or absence of: blepharitis (0.5 points/eye), dermatitis (0.5 for any ear, 1 for tail), swollen/red ankles and paws (0.5 points/limb) and swollen paw/foot digits excluding pollex/hallux (0.1 point/digit). The maximum score possible is 6.1. At the endpoint, lymph nodes were dissected for FACS, one ankle was dissected and skin/toes removed prior to storage in RNA later for qPCR, the other ankle was kept intact and fixed in 10% neutral buffered formalin (HT501128, Sigma). The upper tail, pelvis and distal small intestine were dissected and fixed in formalin.

Disease was induced in B10.RIII mice by hydrodynamic delivery of 6 µg of IL-23 expressing minicircle (System Biosciences) as published (9, 10). Mice were scored every 3 days using the aforementioned SpA scoring system. At one week post-minicircle administration, serum was obtained from saphenous vein and IL-23 assessed by ELISA (BioLegend) to ensure treatment groups had comparable average serum IL-23 concentrations. Tissue was harvested as above for respective analyses.

Intradermal IL-23 induced inflammation was performed as previously reported (11). Mice were first anaesthetized with isoflurane prior to dorsal intradermal injection of 400 ng IL-23 in 20 µl PBS using a 31 gauge, 0.3 ml insulin syringe. For assessment of cytokine in whole ear, ears were snap frozen in liquid nitrogen prior to homogenization with protease cocktail (1 mM Benzamidine hydrochloride, 1 mM PMSF, 1 µg/ml pepstatin, 0.5 µg/ml aprotinin, 0.5 µg/ml leupeptin). IL-17A assessed in ear homogenate with the Luminex assay (R&D Systems). For assessment of skin cells by FACS, mice were first treated with 300 µg BFA (Sigma) in 600 µl PBS for 5 hours prior to tissue harvest. Ears were digested in RPMI with penicillin/streptomycin, 0.25 mg/ml Liberase TM (Roche) and 1 µl/ml BFA (BioLegend) for 60 mins at 37°C in a shaking incubator. After incubation, digested tissue was titrated with a 21 gauge needle, prior to 35 µm filtration. Washed cells were treated with 0.2 mg/ml DNAse in RPMI for 5 minutes at room temperature to obtain single cell suspension for FACS.

JAK kinase radiometric assay

Peptide substrate was prepared in reaction buffer (20 mM HEPES, pH 7.5, 10 mM $MgCl_2$, 1 mM EDTA, 0.02% Brij35, 0.02 mg/mL BSA, 0.1 mM Na_3VO_4, 2 mM DTT, 1% DMSO). TYK2, JAK1, JAK2, or JAK3 (Invitrogen) kinase added, with compounds in DMSO. [^{33}P]ATP added to initiate the reaction with 10 µM

of ATP. The kinase reaction was incubated for 2 hours at room temperature and reactions spotted onto P81 ion exchange paper (Whatman), and then washed extensively in 0.75% phosphoric acid, prior to reading the radioactivity counts.

In vitro murine and human assays

For cell-based JAK activity assays, human PBMCs were pre-incubated with NDI-031407 for one hour in RPMI containing 10% fetal bovine serum, at 37°C in humidified incubator with 5% CO_2. The mixture was then stimulated with IL-12 (R&D Systems) for 30 minutes or GM-CSF (R&D Systems) for 10 minutes. The stimulation was terminated by adding cold PBS and pelleting the cells. Meso Scale Discovery (MSD) lysis buffer was then added to the cell pellets and the lysate analyzed for phospho- and total-STAT4 (for IL-12) or STAT5 (for GM-CSF) per manufacturer's instruction and read on MSD Sector Imager 6000. For the NK-92 cell assay, cells cultured in αMEM without ribonucleosides (STEMCELL Technologies) with 2mM L-glutamine, 0.2 mM myo-inositol (Sigma), 0.1 mM 2-mercaptoethanol, 0.02 mM folic acid (Sigma), 12.5 % horse serum (Sigma), 12.5% heat inactivated Fetal Calf Serum (Gibco) and 100 U/ml recombinant IL-2 (R&D System). For assay, cells were IL-2 starved overnight and NDI-031407 was pre-incubated with the cells for one hour following by IL-12 stimulation for 24 hrs. At the end of incubation cells were pelleted and IFNγ in the supernatant was measured by human IFNγ MSD kit per manufacturer's instructions.

For human in vitro Th17 assays, PBMC were obtained from blood as mentioned above and used fresh. For supernatant cytokine IL-17A detection by ELISA (BioLegend), whole CD4+ T cells were obtained by magnetic selection (Mojosort, BioLegend). Cells were then cultured in XVIVO15 serum-free media (Lonza) with anti-CD2/CD3/CD28 beads (Miltenyi) at a 1 bead: 2 cell ratio, supplemented with 200 U/ml IL-2 (Genscript) for four days. Where indicated cells were further treated with 20 ng/ml IL-1β, 20 ng/ml IL-6 and/or 20 ng/ml IL-23 (all from BioLegend) for the duration of the experiment. Tofacitinib (Selleck Chem), ruxolitinib (Selleck Chem) and NDI-031407 were prepared in DMSO at the indicated concentrations and added for the duration of the experiment. For proliferation and cell viability assessment, cells were stained with Tag-it Violet (BioLegend) before stimulation, then stained at endpoint with Helix NIR and Annexin V FITC (BioLegend) for FACS analysis. For STAT phosphorylation assays, whole PBMC were activated with anti-CD2/CD3/CD28 beads for four days in complete RPMI. Activated cells were then rested in serum-free RPMI for 3 hours, with fixable live/dead staining added in the final 15 mins. Cells were washed in serum free media and pre-treated with JAKinibs at the indicated concentrations for 30 minutes prior to stimulation with 400 ng/ml IL-23 or IL-6 for 15 minutes. H_2O_2 activated pervanadate (P0758S, NEB) was used as a positive control for STAT phosphorylation. Cells were fixed by addition of PFA (BioLegend) directly to stimulated cells and stained for FACS.

For murine in vitro assays, popliteal, inguinal, lumbar, brachial and cervical lymph nodes were homogenized and pooled. For cytokine assays, lymphocytes were pretreated with brefeldin A (BFA, BioLegend) and JAKinib for 30 minutes prior to a 4.5 hour treatment with 20 ng/ml IL-1β (BioLegend) and/or 20 ng/ml IL-23 (RnD) in complete RPMI. PMA/ionomycin (BioLegend) was used as a positive control.

For pSTAT assays, lymphocytes were rested in serum free media for 3 hours before treating for 15 minutes with 400 ng/ml IL-23 or activated pervanadate as above.

SNP analysis

TYK2 SNPs were assessed with off-the-shelf Taqman assays and assessed using a 7900HT (ABI) as per the manufacturer's recommendations. SKG genotyping was performed using a custom Taqman SNP assay: Forward primer CAGGTGGAGAAGCTCATTGCT, Reverse primer CTGGCCGGAATAGAGTTTGC, VIC probe (SKG) AATGCCCTGTTATGAC, FAM probe (WT) ATGCCCTGGTATGACA.

Gene expression

RNA was extracted from human whole blood as discussed above. RNA extracted from sorted human immune cells with TRIzol (Invitrogen), following the manufacturer's protocol. RNA extracted from whole ankle in TRIzol, with Dounce homogenizer. All RNA measured by Nanodrop and used if 260/280 >1.8.

For both murine and human gene expression assays, RNA was reverse transcribed with Superscript IV pretreated with DNAse (Invitrogen). qPCR was performed with power SYBR green (ABI) on the 7900HT (ABI). Primers were designed in house with Primer Express 3 (ABI) and ordered from ACGT (Toronto). All primers were tested for specificity in silico using primer BLAST (NCBI) and by melt curve analysis. Efficiency was assessed in serially diluted samples and ΔΔCt equation used when primer efficiency was >95%, or Pfaffl equation (12) used for primers with efficiency <95%. At least 5 housekeepers were compared between treatments/patients and controls and the most stable housekeeper gene selected for the respective experiments. Primer list provided in Supplementary Table 4.

Flow cytometry

For all panels, except for phosphoflow experiments as discussed above, single cell suspensions were first stained with a fixable live dead stain in NIR spectrum (L/D NIR (Invitrogen) or FVS780 (BD)) as directed by the manufacturers. Cells were blocked with FcX (BioLegend), prior to staining with surface antibodies. For PrimeFlow experiments, cells were fixed, permeabilized and mRNA probe hybridization carried out following the manufacturer's protocol (eBiosciences). For experiments in which transcription factors were stained, cells were fixed and permeabilized with True-Nuclear kit (BioLegend) as directed. For experiments in which cytokines were stained, cells were fixed with a PFA buffer and permeabilized with intracellular staining buffer (BioLegend) as indicated. BFA (BioLegend) with or without PMA/ionomycin (BioLegend) was used for in vitro stimulations to detect cytokine. For phosphoflow experiments, cells were fixed in PFA and permeabilized with True-Phos perm buffer (Biolegend) as indicated. Antibodies used are listed in Supplementary Tables 5 and 6. Data acquired on Aria III, Canto II or Fortesa x20 (BD) and analyzed with

FlowJo. For cell sorting, cells were sorted into RPMI with 50% FCS on ice. Sorted cells were pelleted and RNA immediately extracted by TRIzol.

In vivo imaging

MRI was performed on isoflurane anaesthetized mice using a Bruker 7T system at 60 μm resolution (STTARR, University Health Network, Toronto). 2D FLASH (4.5 ms echo, 750 ms repetition, 5 averages) was used to acquire T1 weighted scans. 2D rare (factor 8, 48 ms echo, 3600 repetition, 6 averages) was used to acquire T2-weighted scans. 7 slices of 0.5 mm each were acquired in the axial plane, centered on the SIJ and two slices nearest to mid SIJ were scored. Images analyzed with MIPAV.

Bones were dissected at endpoint and fixed in 10% NBF for 3 days prior to storage in PBS. Imaging was performed on Sanco μCT100 with 10 μm resolution (70 kV/114 μA, aluminum filter 0.5 mm and 1200 mgHA/ccm beam hardening) (Sunnybrook Hospital, Toronto). The scans were analyzed with Image J.

Histopathology and scoring

All tissue was fixed in 10% NBF for up to 3 days. Bone tissue decalcified with CalExII (Fisher). Tissue was paraffin embedded prior to sectioning for H&E (TCP, Mt Sinai Hospital, Toronto). Slides scored by single blinded reader. We used published pathologic scoring rubrics for the skin (13), gut (14), ankle (5, 15), tail (5, 16), and SIJ (5). All scoring rubrics given in Supplementary Tables 7-10.

Statistics

All statistical analysis performed with GraphPad Prism. Data tested for normality before statistical test selected. All graphs show mean with standard error of mean, unless indicated otherwise. Where applicable, individual points represent separate patient, animal or treated well. Two-tailed statistical tests used, with specific test indicated in the respective figure legends. For all graphs: A P value less than 0.05 was considered significant. Not significant (n.s.), $p>0.05$; (*) $0.05>p>0.01$; (**) $0.01>p>0.001$; (***) $0.001>p>0.0001$; (****) $p<0.0001$.

1. van der Linden S, Valkenburg HA, Cats A. Evaluation of diagnostic criteria for ankylosing spondylitis. A proposal for modification of the New York criteria. [Internet]. *Arthritis Rheum.* 1984;27(4):361–8.

2. Aletaha D et al. 2010 Rheumatoid arthritis classification criteria: An American College of Rheumatology/European League Against Rheumatism collaborative initiative [Internet]. *Arthritis Rheum.* 2010;62(9):2569–2581.

3. Rudwaleit M et al. The Assessment of SpondyloArthritis International Society classification criteria for

peripheral spondyloarthritis and for spondyloarthritis in general. [Internet]. *Ann. Rheum. Dis.* 2011;70(1):25–31.

4. International League of Associations for Rheumatology. D et al. *Clinical rheumatology. [Internet]*. Springer London; 1999:

5. Ruutu M et al. β-glucan triggers spondylarthritis and Crohn's disease-like ileitis in SKG mice [Internet]. *Arthritis Rheum.* 2012;64(7):2211–2222.

6. Sakaguchi N et al. Altered thymic T-cell selection due to a mutation of the ZAP-70 gene causes autoimmune arthritis in mice [Internet]. *Nature* 2003;426(6965):454–460.

7. Prchal-Murphy M et al. TYK2 Kinase Activity Is Required for Functional Type I Interferon Responses In Vivo [Internet]. *PLoS One* 2012;7(6):e39141.

8. Karaghiosoff M et al. Partial impairment of cytokine responses in Tyk2-deficient mice. [Internet]. *Immunity* 2000;13(4):549–60.

9. Sherlock JP et al. IL-23 induces spondyloarthropathy by acting on ROR-γt+ CD3+CD4−CD8− entheseal resident T cells [Internet]. *Nat. Med.* 2012;18(7):1069–1076.

10. Reinhardt A et al. Interleukin-23-Dependent γ/δ T Cells Produce Interleukin-17 and Accumulate in the Enthesis, Aortic Valve, and Ciliary Body in Mice [Internet]. *Arthritis Rheumatol.* 2016;68(10):2476–2486.

11. Cibrian D et al. CD69 controls the uptake of L-tryptophan through LAT1-CD98 and AhR-dependent secretion of IL-22 in psoriasis [Internet]. *Nat. Immunol.* 2016;17(8):985–996.

12. Pfaffl MW. A new mathematical model for relative quantification in real-time RT-PCR. [Internet]. *Nucleic Acids Res.* 2001;29(9):e45.

13. Baillet AC et al. High *Chlamydia* Burden Promotes Tumor Necrosis Factor-Dependent Reactive Arthritis in SKG Mice [Internet]. *Arthritis Rheumatol.* 2015;67(6):1535–1547.

14. Erben U et al. A guide to histomorphological evaluation of intestinal inflammation in mouse models. [Internet]. *Int. J. Clin. Exp. Pathol.* 2014;7(8):4557–76.

15. Yang YH, Hamilton JA. Dependence of interleukin-1-induced arthritis on granulocyte-macrophage colony-stimulating factor [Internet]. *Arthritis Rheum.* 2001;44(1):111–119.

16. Tseng H-W et al. Inflammation-driven bone formation in a mouse model of ankylosing spondylitis: sequential not parallel processes. [Internet]. *Arthritis Res. Ther.* 2016;18:35.

	AS	HC	RA
sex (M/F)	49/27	29/18	6/15
age (yrs)	42±16	37±12	54±11
HLA-B27+	79%	-	-
CRP	7.6 ±8.7	-	-
ESR	10.2 ±9.7	-	-
BASDAI	3.7 ±2.44	-	-
rs34536443 (CC/CG/GG)	0/1/75	0/0/47	0/0/16
rs1272056 (CC/AC/AA)	1/22/53	0/4/43	0/1/16
rs35164067 (AA/AG/GG)	8/23/45	3/19/25	1/5/11

Supplementary Table 1: Clinical information on *TYK2* primeflow, whole blood qPCR and PMA/ionomycin stimulated cohort. Age, CRP, ESR and BASDAI presented as mean +/- SD. CRP measured in mg/L and ESR in mm/hr.

	SpA	RA
sex (M/F)	17/10	11/21
age (yrs)	43(31-54)	60(53-75)
disease duration (yrs)	3 (1-10)	2.5 (1-12)
swollen joint count	1(1-2)	2(1-11)
tender joint count	2(1-4)	5(2-18)
CRP	9.6 (1.25-75)	19.75 (2.8-43.7)
ESR	12 (3.5 – 39)	30.6 (11-45)
DMARDs	59%	62.5%
biologics	7.4%	3.1%
axial involvement	67%	N/A

Supplementary Table 2: Clinical information for synovial biopsies. Values presented as average with range where applicable. CRP measured in mg/L and ESR in mm/hr.

	non-progressors	progressors
sex (M/F)	55/24	71/14
HLA-B27+	80%	74%
age at last x-ray (yrs)	38 ±13	51 ±11
baseline CRP	11.9 ±23	20.5±24
baseline BASDAI	4.6 ±2.7	5.05 ±2.4
TNFi	65%	69%
grade 3-4 sacroilitis	80%	94%
duration between x-rays (yrs)	5 ±1.2	5 ±2.3
baseline mSASSS	3 ±11	17 ±19
mSASSS/yr	0.00	2.05 ±1.7

Supplementary Table 3: Clinical information for AS progressor/non-progressor cohort. Age, CRP, ESR and BASDAI presented as mean +/- SD. CRP measured in mg/L and ESR in mm/hr. All AS patients have at least grade 1-2 sacroiliitis.

Species	Gene	Forward	Reverse
Human	*TYK2*	GTCTGCATCCACATTGCACAT	GAGGGCAAAGAGATTGAAGCA
Human	*HPRT*	TTATGGACAGGACTGAACGTCTTG	GCACACAGAGGGCTACAATGTG
Human	*GAPDH*	GCTCTCTGCTCCTCCTGTTC	CGCCCAATACGACCAAATCC
Mouse	*Il23a*	CCAGCGGGACATATGAATCTACT	TCACAACCATCTTCACACTGGAT
Mouse	*Il23r*	CACTGCTGAATGTCCTGGTCAT	GGTGGATGTCCAGAGGAAATGT
Mouse	*Il22*	TCGTCAACCGCACCTTTATG	CCGGACATCTGTGTTGTTATCTG
Mouse	*Il17a*	TCTGTGTCTCTGATGCTGTTGCT	TCGCTGCTGCCTTCACTGTA
Mouse	*Tyk2*	GCATCAGACCCCACTGTTTTC	AAGTGACCCTCACCCAAATCC
Mouse	*Rpl4*	TTGGCGGAAGGCTGCTT	CGGTGTTCATCATCTTGTGCAT

Supplementary Table 4: primer list for qPCR.

Antigen	Fluorochrome	Clone	Company
CCR7	PE.Dazzle	G043H7	BioLegend
CD14	APC	HCD14	BioLegend
CD161	BV711	DX12	BD Biosciences
CD19	FITC	4G7	BioLegend
CD3e	BV605	OKT3	BioLegend
CD3e	APC	145-2C11	BD Biosciences
CD4	BUV395	RPA-T4	BD Biosciences
CD4	PE	H129.19	BD Biosciences
CD45RA	BV421	HI100	BD Biosciences
CD45RA	BV605	HI100	BioLegend
CD56	BUV737	NCAM162	BD Biosciences
CD8a	FITC	RPA-T8	BioLegend
CD8a	AF700	53-6.7	BioLegend
IFNy	PE	4S.B3	BioLegend
IL-17A	AF647	BL168	BioLegend
pSTAT3	BV421	13A3-1	BioLegend
pSTAT4	AF488	38/p-STAT4	BD Biosciences
pSTAT5	PE.Cy7	47/Stat5(pY694)	BD Biosciences
TCRab	PB	IP26	BioLegend
TCRyd	FITC	B1	BioLegend
TNFa	PE.Dazzle	MAb11	BioLegend
Va7.2	PE.Cy7	3C10	BioLegend

Supplementary Table 5: anti-human antibodies used.

Antigen	Fluorochrome	Clone	Company
CCR6	APC	29-2L17	BioLegend
CD25	BV605	BC96	BioLegend
CD25	BV605	BC96	BioLegend
CD278 (ICOS)	PB	C398.4A	BioLegend
CD278 (ICOS)	BV785	C398.4A	BioLegend
CD279 (PD-1)	PerCP.Cy5.5	29F.1A12	BioLegend
CD3e	BUV737	145-2C11	BD Biosciences
CD3e	PerCP.Cy5.5	145-2C11	BioLegend
CD3e	APC	145-2C11	BD Biosciences
CD4	BUV395	GK1.5	BD Biosciences
CD45	PB	30-F11	BioLegend
CD8a	PE.Cy7	53-6.7	BioLegend
FoxP3	PB	MF-14	BioLegend
IFNy	PB	4S.B3	BioLegend
IL-10	PE.Cy7	JES5-16E3	BioLegend
IL17A	BV605	TC11-18H10.1	BioLegend
IL-17F	PE.CF594	O79-289	BD Biosciences
IL-22	PerCP.Cy5.5	Poly5164	BioLegend
IL-22	APC	Poly5164	BioLegend
Ki67	AF488	B56	BD Biosciences
PLZF	PE	9.00E+12	BioLegend
pSTAT3	BV421	13A3-1	BioLegend
RORyt	PE	Q21-559	BD Biosciences
RORyt	AF647	Q21-559	BD Biosciences
Tbet	APC	4B10	BioLegend
TCRb	BUV737	H57-597	BD Biosciences
TCRb	PE	H57-597	BioLegend
TCRyd	BV605	GL3	BioLegend
TCRyd	AF488	GL3	BioLegend
TNFa	FITC	mP6-XT22	BioLegend

Supplementary Table 6: anti-mouse antibodies used.

Location	Feature	Score	Extent	Description
Stratum corneum	hyperkeratosis or parakeratosis	0	normal	
		1	present	
Epidermis	thickening	0	normal	1-2 layers keratinocytes
		1	mild	2-4 layers keratinocytes
		2	moderate	4-6 layers keratinocytes
		3	servere	6+ layers keratinocytes
Dermis	WBC infiltration	0	normal	
		1	mild	some immune cells, <1/3 increase of normal thickness
		2	moderate	immune cells common, 1/3 - 2/3 increase of normal thickness
		3	severe	massive WBC infiltrate, >2/3 increase of normal thickness

Supplementary Table 7: Histopathology scoring rubric for skin.

Location	Feature	Score	Extent	Description
Global architecture	ulceration	0	none	
		1	present	
	villi	0	normal	crypt:length ratio >2:1
		1	mild	slight villous blunting (crypt:length ratio 1:1-2:1)
		2	severe	villous blunting or atrophy
	granulomas	0	absent	
		1	present	
Epithelium	hyperplasia	0	normal	
		1	minimal	<25%, disperse enhanced mitosis
		2	mild	25-50%, mitosis in middle of crypt epithelium, far from crypt base
		3	severe	>50%, mitosis in upper third of crypt epithelium, far from crypt base
	goblet cell	0	normal	normal mucin, >50 goblet cells/field @ 40x
		1	minimal	mild mucin loss, 25-50 goblet cells/field @ 40x
		2	mild	moderate loss mucin, 10-25 goblet cells/field @ 40x
		3	severe	severe loss mucin, <10 goblet cells/field @40x
	cryptitis	0	absent	normal
		1	present	neutrophils between crypt epithelium or in lumen
Lamina propria	WBC infiltrate - severity	0	normal/minimal	WBC<10% area
		1	mild	10-25% area, some neutrophils
		2	moderate	25-50% area
		3	severe	>50% area, dense WBC infiltrate
	WBC infiltrate - extent	0	normal	
		1	mild	lamina propria
		2	severe	lamina propria and muscularis mucosa

Supplementary Table 8: Histopathology scoring rubric for gut tissue.

Location	Feature	Score	Extent	Description
Soft tissue	synovium	0	normal synovial layer	intact, monolayer epithelial cells
		1	mild synovitis	patchy hyperplasia/loss epithelium
		2	severe synovitis	widespread hyperplasia and immune infiltrate of synovial layer
	fat pad/joint capsule	0	normal	none/normal
		1	mild	patchy WBC infiltrate
		2	severe	severe/widespread with loss tissue architecture (>50% soft tissue is WBC infiltrate)
	pannus	0	none	
		1	present	
	joint space	0	normal	clear joint space
	(synovial cavity/bursa)	1	mild	some WBC and/or fibrous material, but still clear spaces
		2	severe	excessive WBC/fibrous material (>50% of cavity)
Hard tissue	cartilage	0	normal	
		1	mild	patchy loss of cartilage, especially at edges
		2	severe	excessive loss (>50%)
	bone	0	normal	no subchrondral infiltrate, no destruction
		1	mild	some infiltrate, possible remodelling
		2	severe	severe infiltrate (granulomatous), large areas bone remodelling
Tendon/ligament	Achilles and/or	0	normal	normal tendon/ligament
	plantar fascia	0.5	mild	mild inflammation at insertion or along side of tendon/ligament
		1	mild-infiltrates	mild inflammation at insertion which continues up tendon/ligament
		1.5	severe	severe inflammation with bone involvment
		2	severe - destruction	severe inflammation with loss of normal insertion interface

Supplementary Table 9: Histopathology scoring rubric for ankle.

Location	Feature	Score	Extent	Description
Disc		0	normal	
		1	mild	<50% destruction
		2	severe	>50% destruction
Hard tissue	cartilage	0	normal	
		1	mild	some loss of articular cartilage/growth plate cartilage
		2	severe	excessive loss (>50%)
	bone	0	normal	
	(erosion)	1	mild	some bone loss
		2	severe	large areas bone remodelling/destruction
	bone	0	normal	
	(edema)	1	mild	some areas of cell infiltrate (<50%)
		2	severe	extensive cell infiltrate (>50%)
Ligament	Intervertebral	0	normal	normal ligament
		1	mild	some inflammation/cell infiltrate along ligment
		2	severe	extensive inflammation with loss of normal insertion interface

Supplementary Table 10: Histopathology scoring rubric for vertebrae (tail).

Location	Feature	Score	Extent	Description
SIJ	joint space area	0	normal	definitive gap seen between sacrum and ileum
		1	narrowing	patchy loss of gap
		2	fusion	no gaps seen
	joint space infiltrate	0	normal	
		1	mild	some infiltrate seen in joint space
		2	severe	excessive infiltrate
	cartilage	0	normal	
		1	mild	some loss of articular cartilage/growth plate cartilage
		2	severe	excessive loss (>50%)
	Bone erosion	0	normal	
		1	mild	some bone loss
		2	severe	large areas bone remodelling/destruction
	sacral ligament infiltrate	0	normal	absent
		1	mild	patchy
		2	severe	severe
Bone	vascularization	0	normal	few defined blood vessels (not sinusoids) (<10/20x field of view)
(non-SIJ)		1	mild	definied blood vessels (10-20/20x view)
		2	severe	large increase in blood vessels (>20/20x view)
	bone loss	0	normal	cortex intact and thick (>30% 20x field of view)
		1	mild	damage (pits) or thining of cortex (15-30% 20x field of view)
		2	severe	extensive degredation or thinning of cortex (<15% 20x field of view)
	bone edema	0	normal	vissible sinusoids/open spaces
		1	mild	patchy empty spaces, increased cellularity
		2	severe	extensive cellularity/loss of sinusoids
Facet joint	infiltrate	0	normal	absent
		1	mild	patchy
		2	severe	severe

Supplementary Table 11: Histopathology scoring rubric for pelvis.

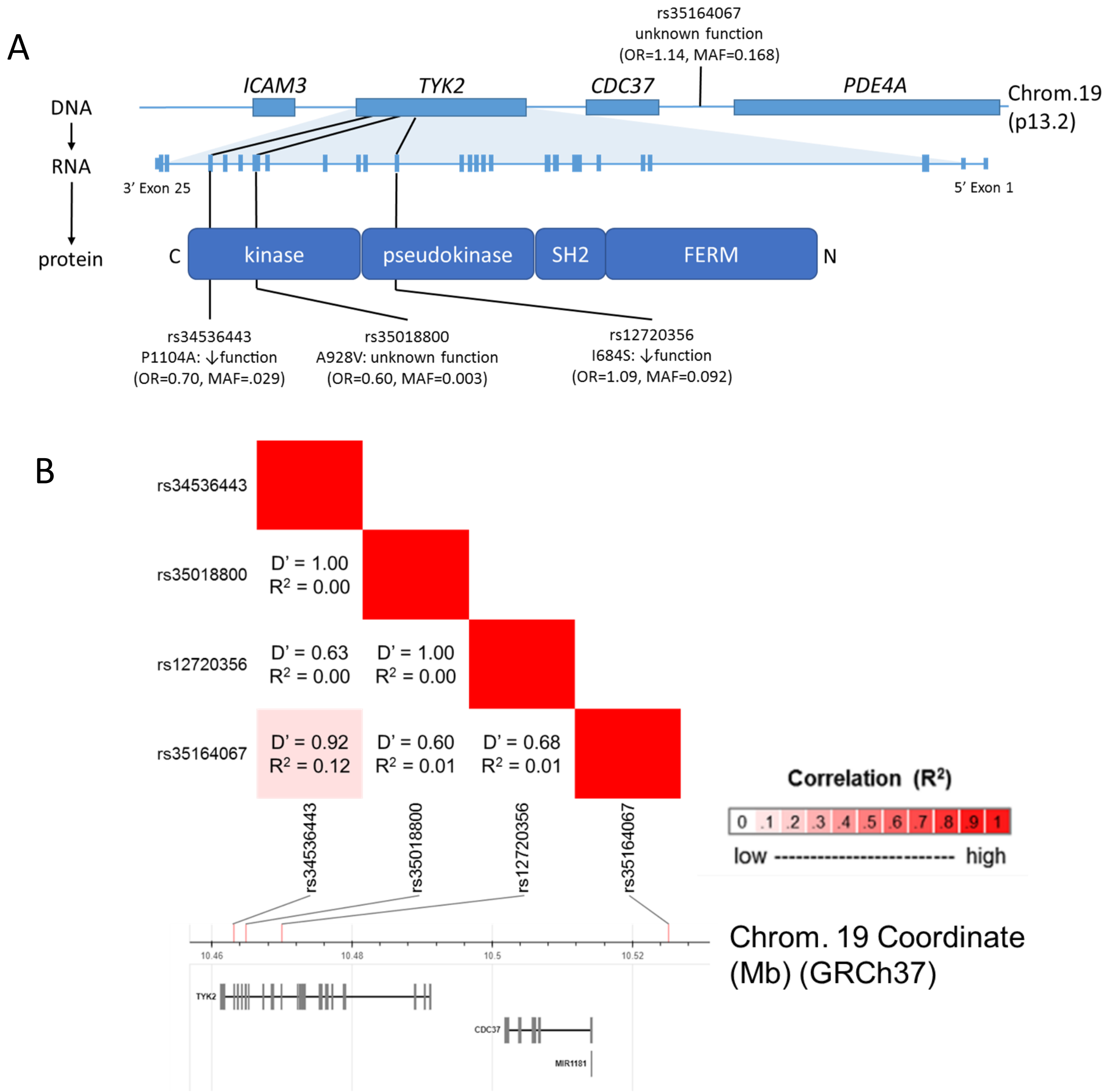

Supplementary Figure 1: AS associated *TYK2* risk SNPs are in linkage disequilibrium but are poorly correlated. A) Schematic of TYK2 gene and protein, showing the locations of AS-associated *TYK2* SNPs. Odds ratios (OR) are from published GWAS. Minor allele frequencies (MAF) are from Caucasian populations reported in the 1000 Genomes Project. Function of minor allele annotated where known. B) LD data generated from LDlink tool (analysistools.nci.nih.gov/LDlink/), in Caucasian populations from the 1000 genomes project.

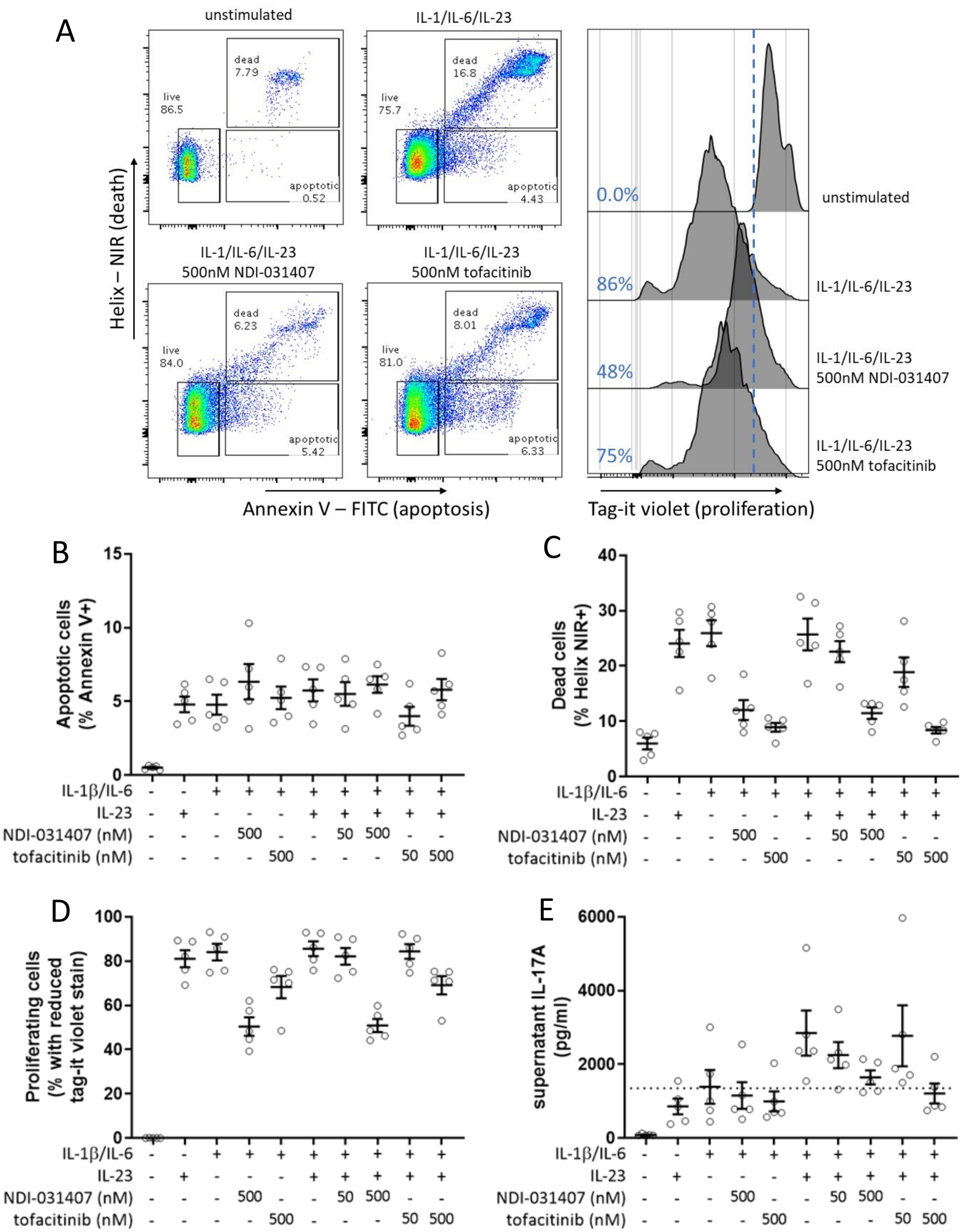

Supplementary Figure 2: Extended data for human CD4+ T cell *in vitro* assays. Magnetically purified whole CD4+ T cells from 5 healthy individuals stained with tag-it violet before culture with anti-CD2/CD3/CD28 beads and IL-2 for 3 days with NDI-031407 or tofacitinib in the presence of 20 ng/ml of the indicated cytokines. Unstimulated wells cultured without TCR stimulation or cytokines. A) Representative FACS plots demonstrating gating strategy used to identify B) apoptotic, C) dead and D) live proliferating CD4+ T cells. E) IL-17 in endpoint supernatants measured by ELISA. Each datapoint is independent donor. Experiment performed once.

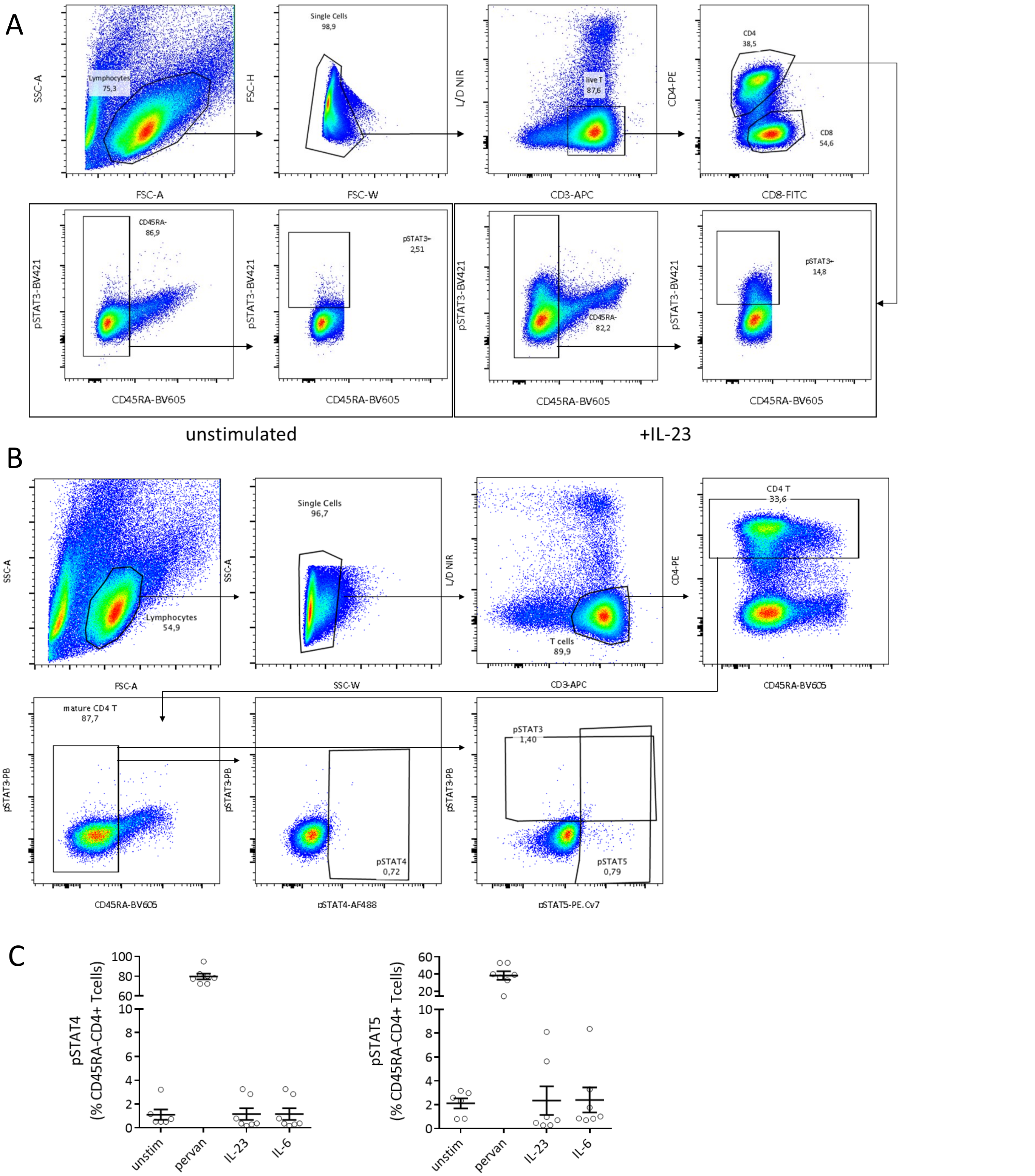

Supplementary Figure 3: Extended data for human pSTAT experiments. A) Gating strategy for pSTAT3 (Y705) experiments in Figure 1C-D. B) Gating strategy for pSTAT3/4/5. C) Graphs showing pSTAT4 (Y693) and pSTAT5 (Y694) staining under the indicated stimulations. Pervan = pervanadate. Each data point is independent donor. pSTAT4/pSTAT5 experiment performed once.

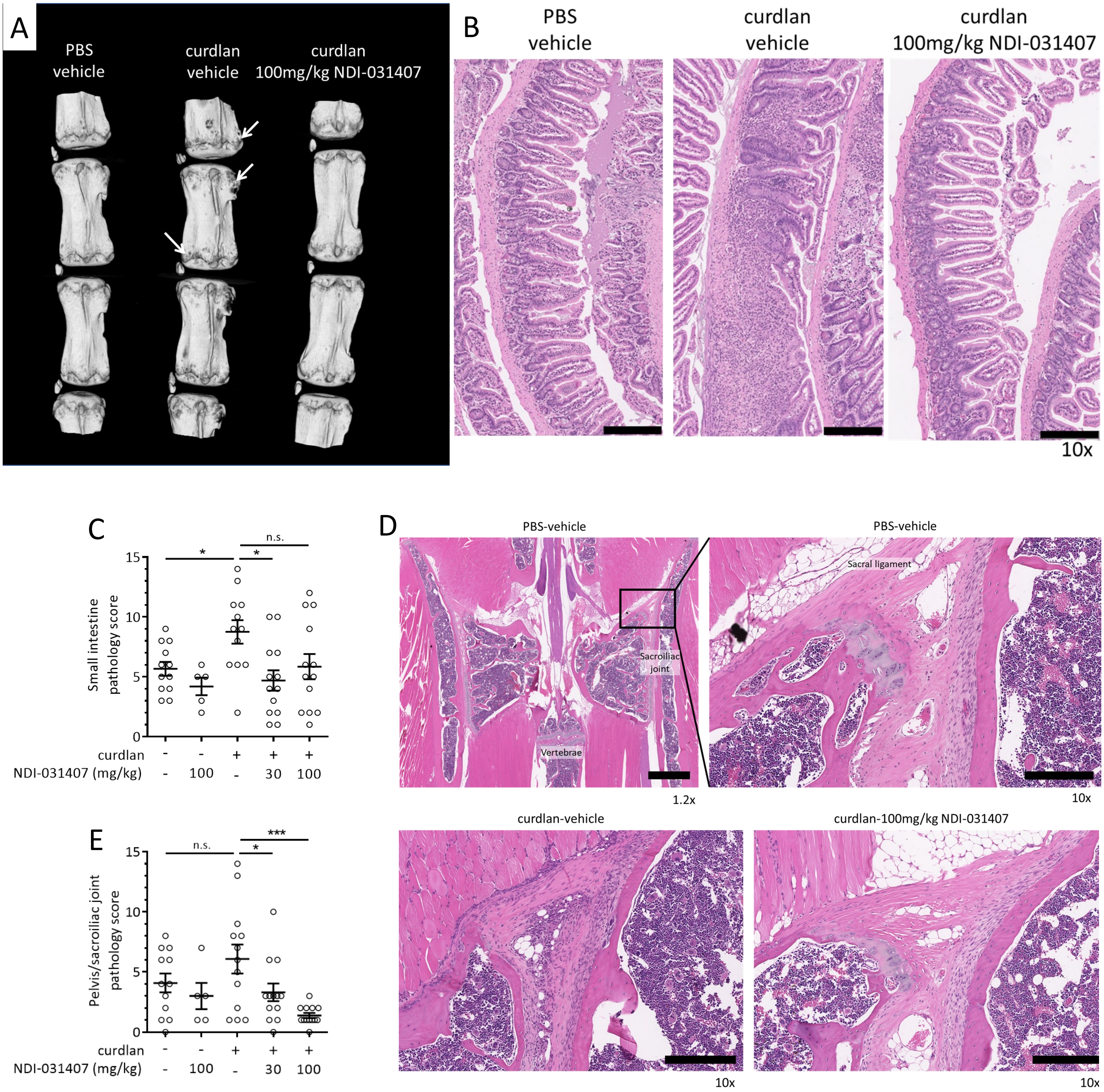

Supplementary Figure 4: Extended data of disease progression in SKG mice with TYK2 inhibition. A) µCT performed on the tail of mice from the indicated treatment groups. Arrows indicate sites of erosion at intervertebral ligament insertions. H&E images from animals representative of the indicated treatment groups from B) the small intestine and D) sacroiliac joint. For 1.2x magnified image, scale bar is 1 mm, for 10x images, scale bar is 200 µm. Pathology scores for C) small intestine and E) SIJ pooled from 5-13 mice/group. Disease-free vs curdlan/vehicle animals analyzed by unpaired T-test and NDI-031407 treated animals compared vehicle treated with one-way ANOVA with Dunnet's post-test. Each data-point is from a single mouse.

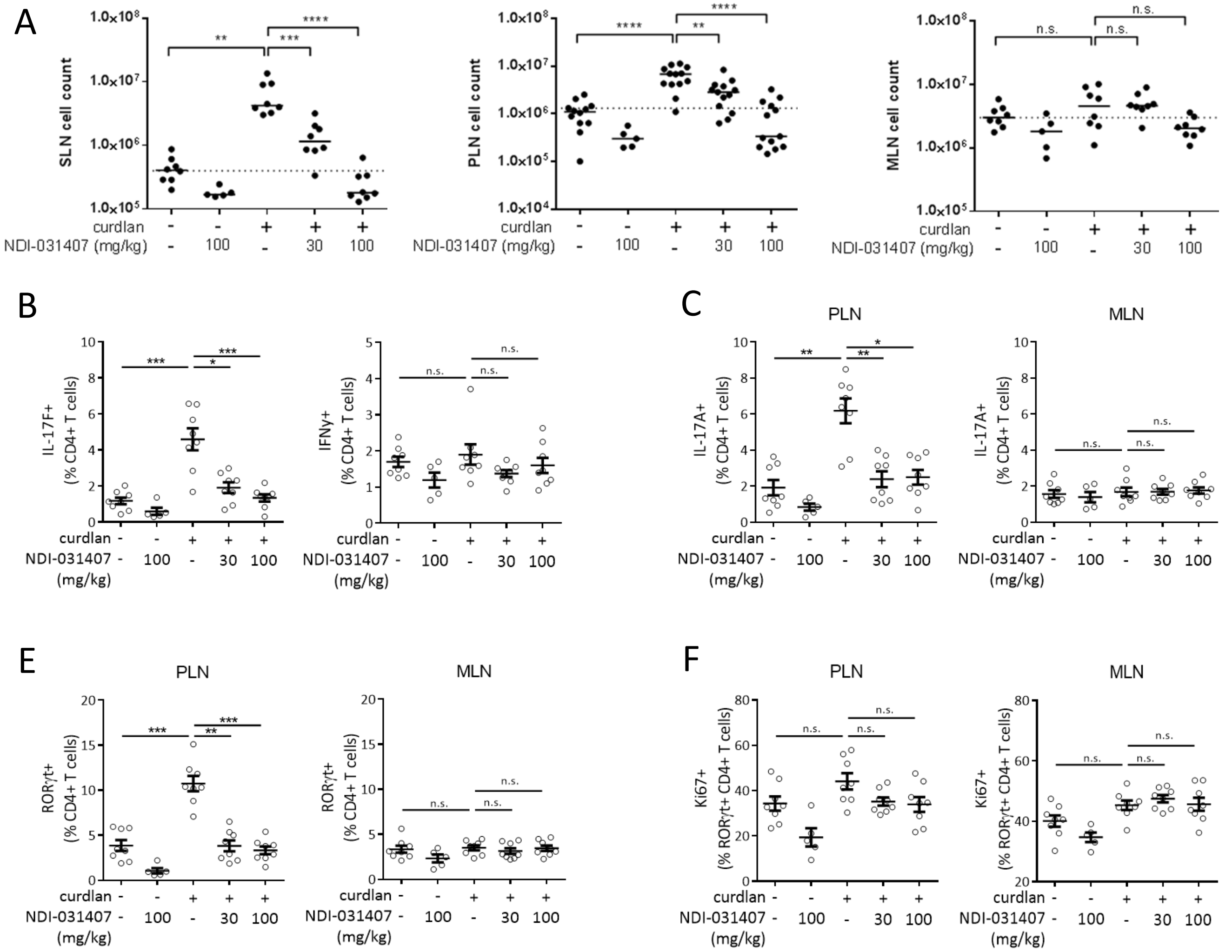

Supplementary Figure 5: Lymph node counts and extended flow cytometry data in NDI-031407-treated SKG mice. A) Lymph node counts by trypan blue from the indicated treatment groups. Hashed line set at average cell count for disease-free, vehicle treated mice. B) IL-17F and IFNγ expression in SLN. C-F) IL-17A, RORγt and Ki67 expression in PLN and MLN for comparison to SLN data in Figure 4. SLN = sciatic lymph node, PLN = popliteal lymph node, MLN = mesenteric lymph node. For all graphs, disease-free mice compared to curdlan/vehicle treated mice with Mann-Whitney test. Curdlan/NDI-031407 treated mice compared to curdlan/vehicle controls with Kruskal-Wallis test with Dunn's posttest. Each data point is from a single mouse.

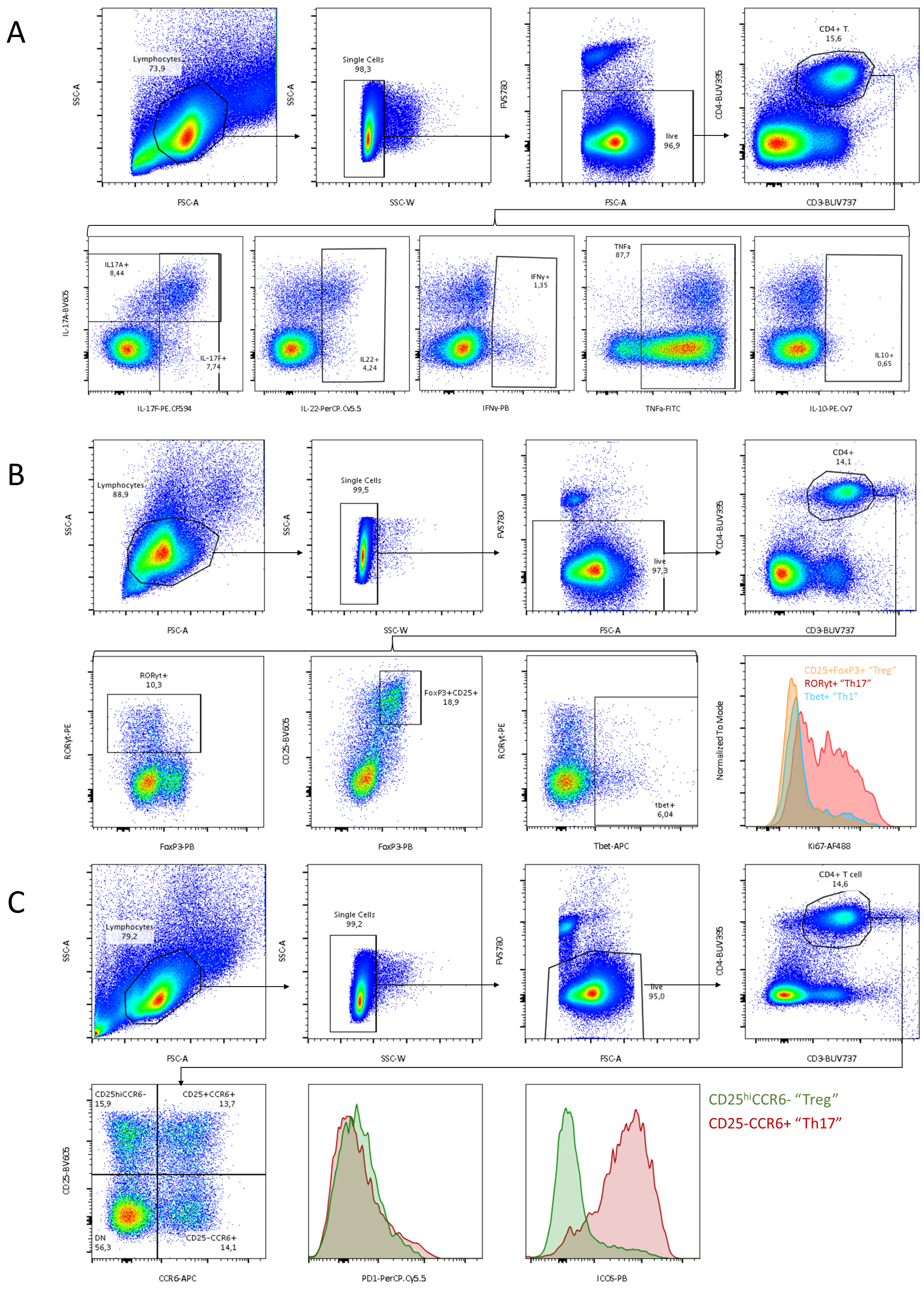

Supplementary Figure 6: Extended data for flow cytometry in NDI-031407-treated SKG mice. Representative gating strategies for flow cytometry panels from curdlan/vehicle-treated in popliteal lymph nodes. A) Intracellular flow cytometry in PMA/ionomycin re-stimulated cells. B) Transcription factor staining in unstimulated cells. C) surface staining for activation markers in unstimulated cells.

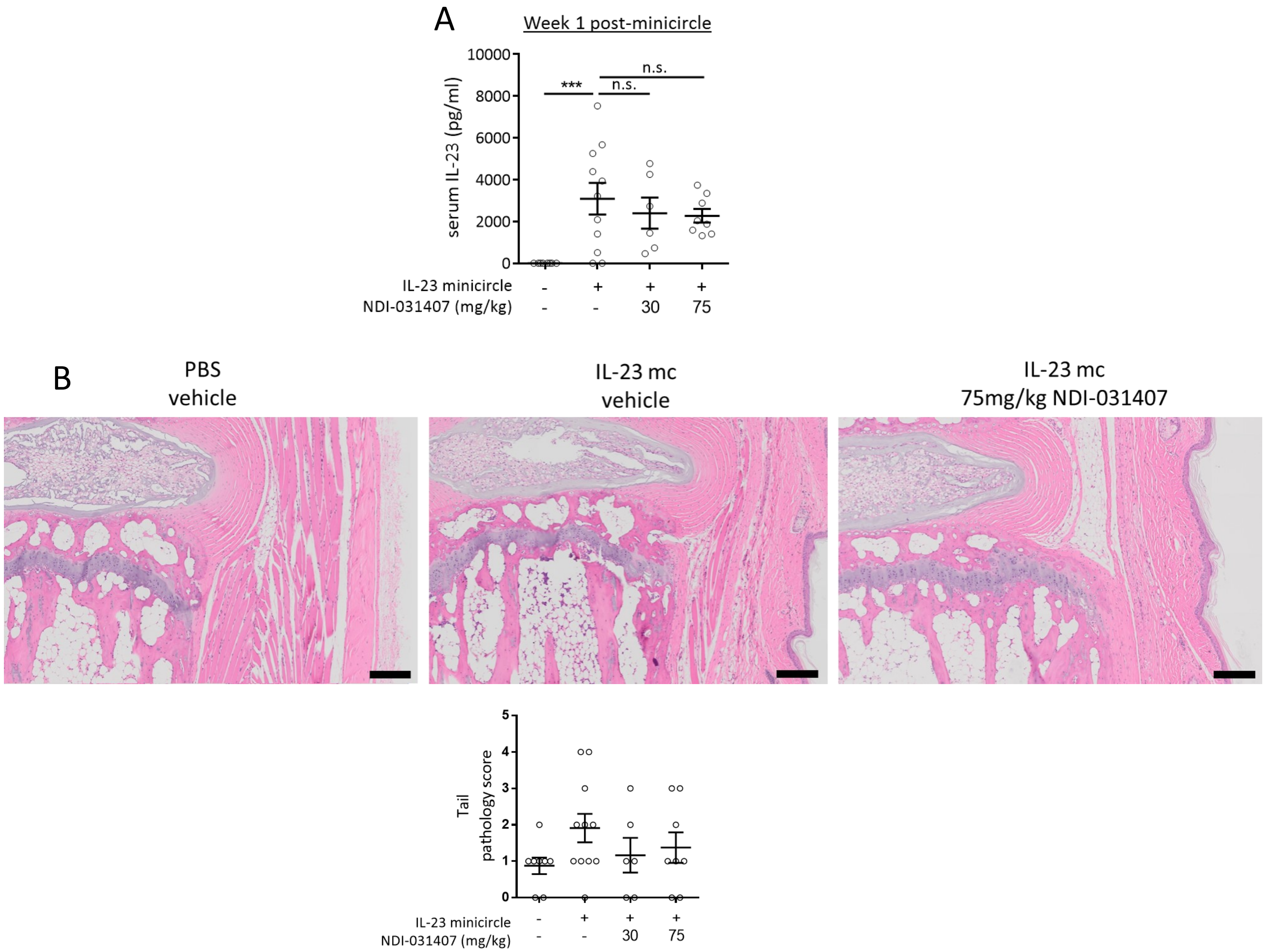

Supplementary Figure 7: Extended data for IL-23 minicircle model of SpA. IL-23 minicircle or PBS administered by hydrodynamic delivery. A) Serum IL-23 assessed by ELISA at one week post-minicircle. Mice divided into treatment groups with comparable average serum IL-23 levels. B) Representative images and pooled data from H&E stained tail vertebrae in the IL-23 minicircle model of SpA. Tissue obtained at endpoint (3 weeks post-IL-23 minicircle). Scale bar is 200 µm. Disease-free mice compared to curdlan/vehicle treated mice with Mann-Whitney test. Curdlan/NDI-031407 treated mice compared to curdlan/vehicle controls with Kruskal-Wallis test with Dunn’s posttest. Each datapoint is a single mouse.

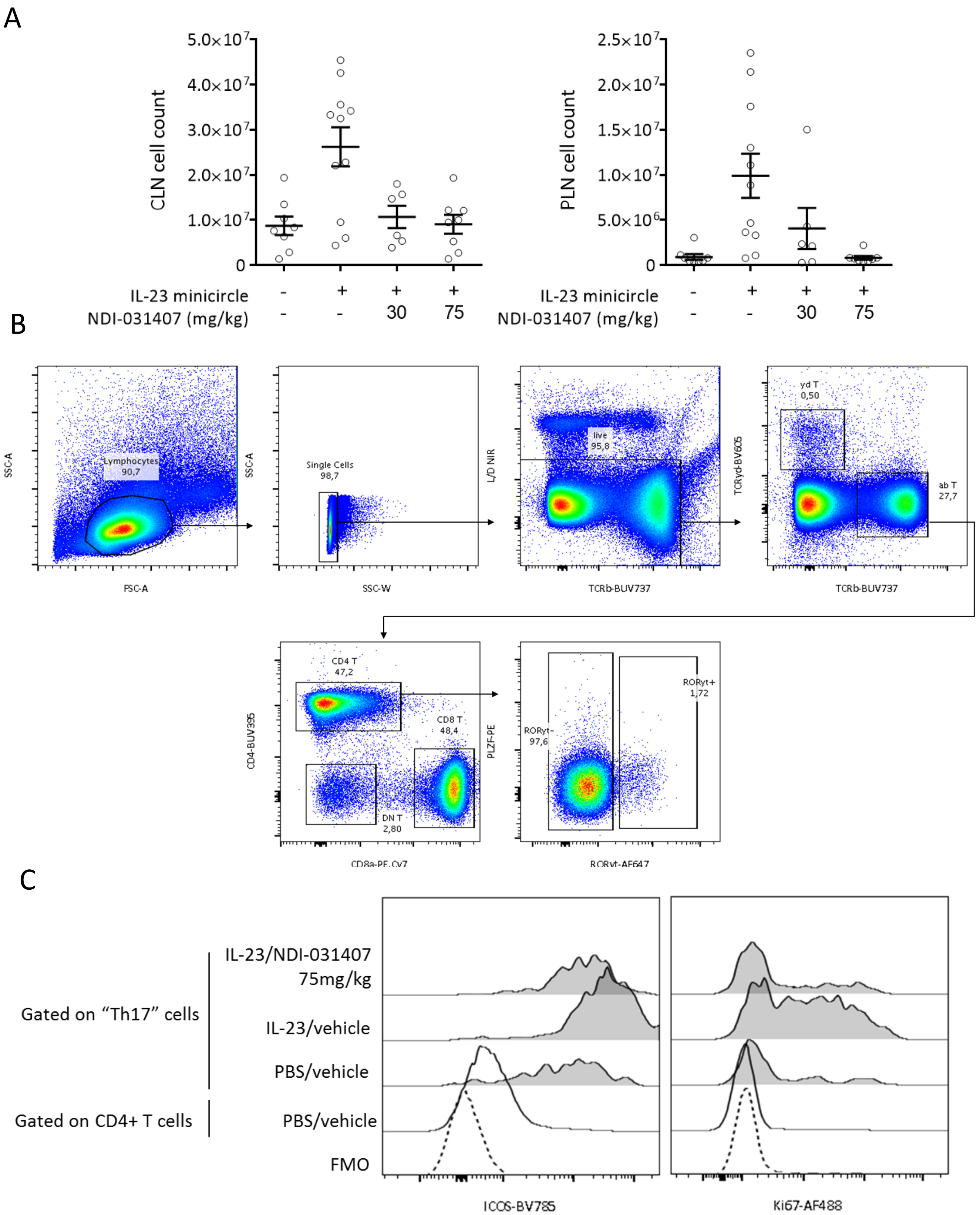

Supplementary Figure 8: Lymph node counts and extended FACS data for IL-23 minicircle experiments. A) Lymph node cell counts at 3 weeks post IL-23 mincircle. B) Representative plots from PLN to demonstrate gating strategy for T cell populations. Th17 cells defined as RORγt+CD4+TCRβ+ lymphocytes. C) ICOS and Ki67 staining on the indication cell populations. FMO is gated on CD4+TCRb+RORyt+"Th17" cells. Each datapoint is a single mouse.

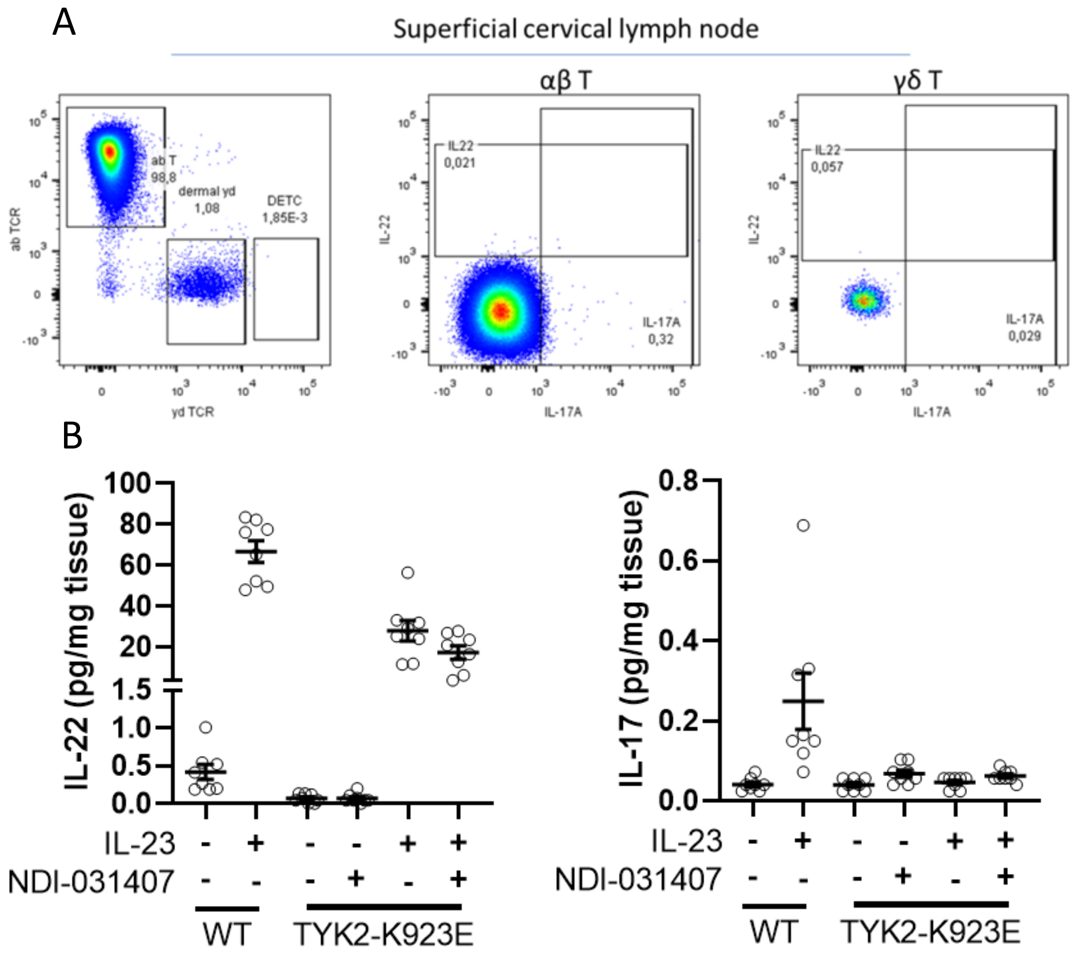

Supplementary Figure 9: Extended data for model of local IL-23 inflammation. A) IL-23 and PBS delivered intradermally to contralateral ears in a vehicle/Brefeldin A treated mouse from the experiment depicted in Figure 5. Draining lymph node (CLN) homogenates were tested by flow cytometric analysis for the indicated cytokines in T cells. Experiment repeated in NDI-031407-treated and TYK2^{K923E} mice with similar results (not shown). B) C57BL/6 (WT) or TYK2^{K923E} mice injected intradermally with PBS or IL-23 as above. 100mg/kg NDI-031407 or methylcellulose oral BID started one day prior to the first IL-23 injection and continued daily. Ears harvest 5 hr after final IL-23 injection for cytokine analysis by luminex assay as discussed in the methods. Each datapoint is a single mouse.

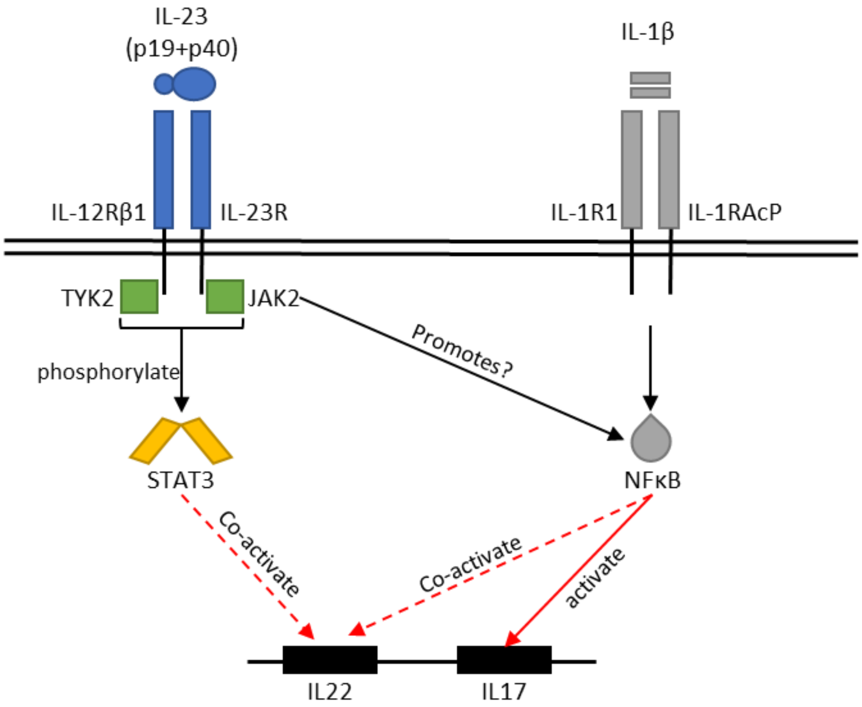

Supplementary Figure 10: Conceptual summary of IL-23R signaling in the induction of IL-22 and IL-17A.

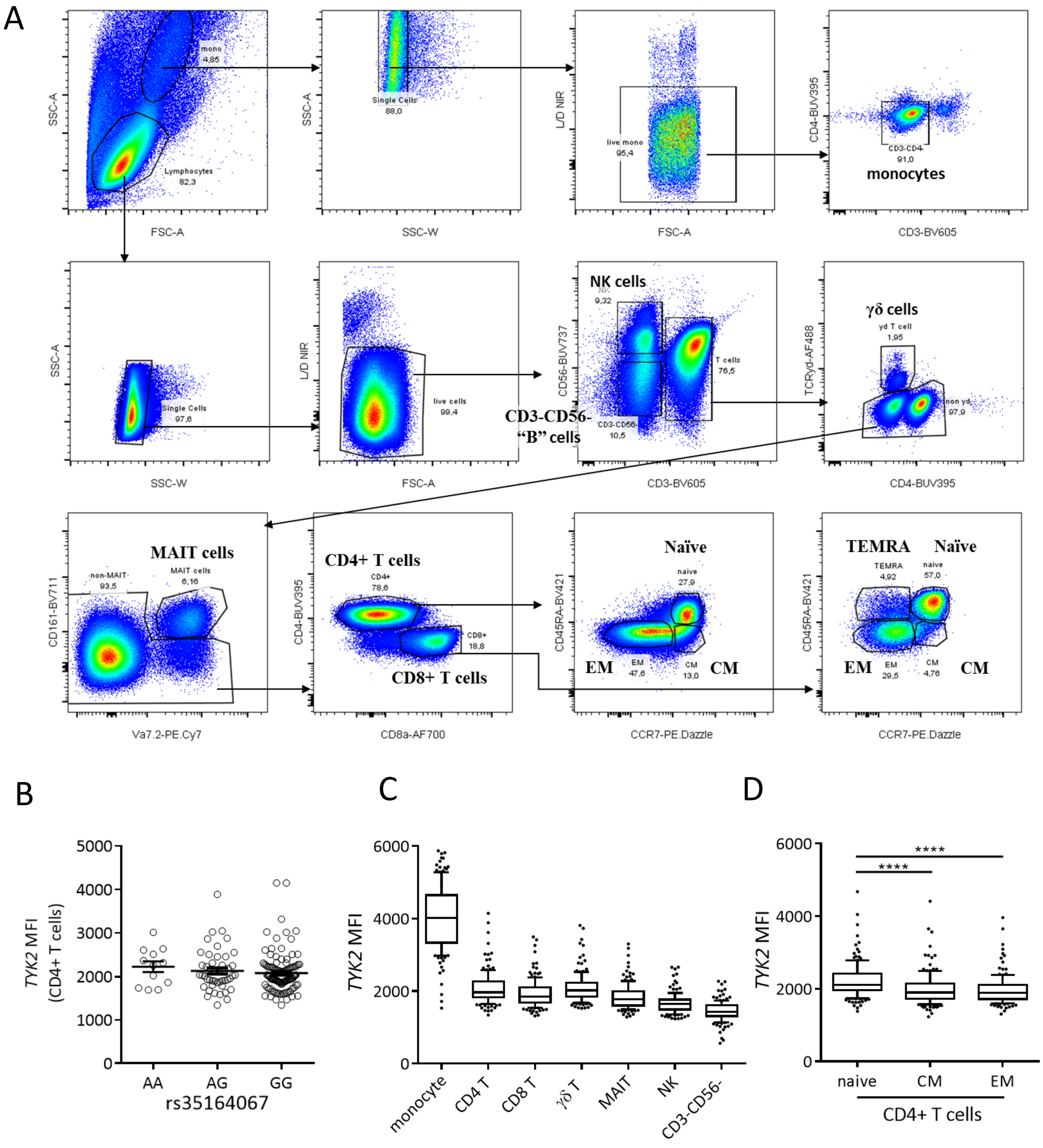

Supplementary Figure 11: Extended data for *TYK2* PrimeFlow FACS. A) Gating strategy for identification of major cell populations. B) CD4+ T cell *TYK2* expression in the whole cohort (AS/RA/HC) stratified by rs35164067. *TYK2* expression in whole cohort (AS/RA/HC) by C) broad cell type and D) CD4+ T cell subset. C and D displayed as box and whisker plots with whiskers at 10th-90th percentiles and assessed by one-way ANOVA with Tukey post-test. Each datapoint is an independent donor.

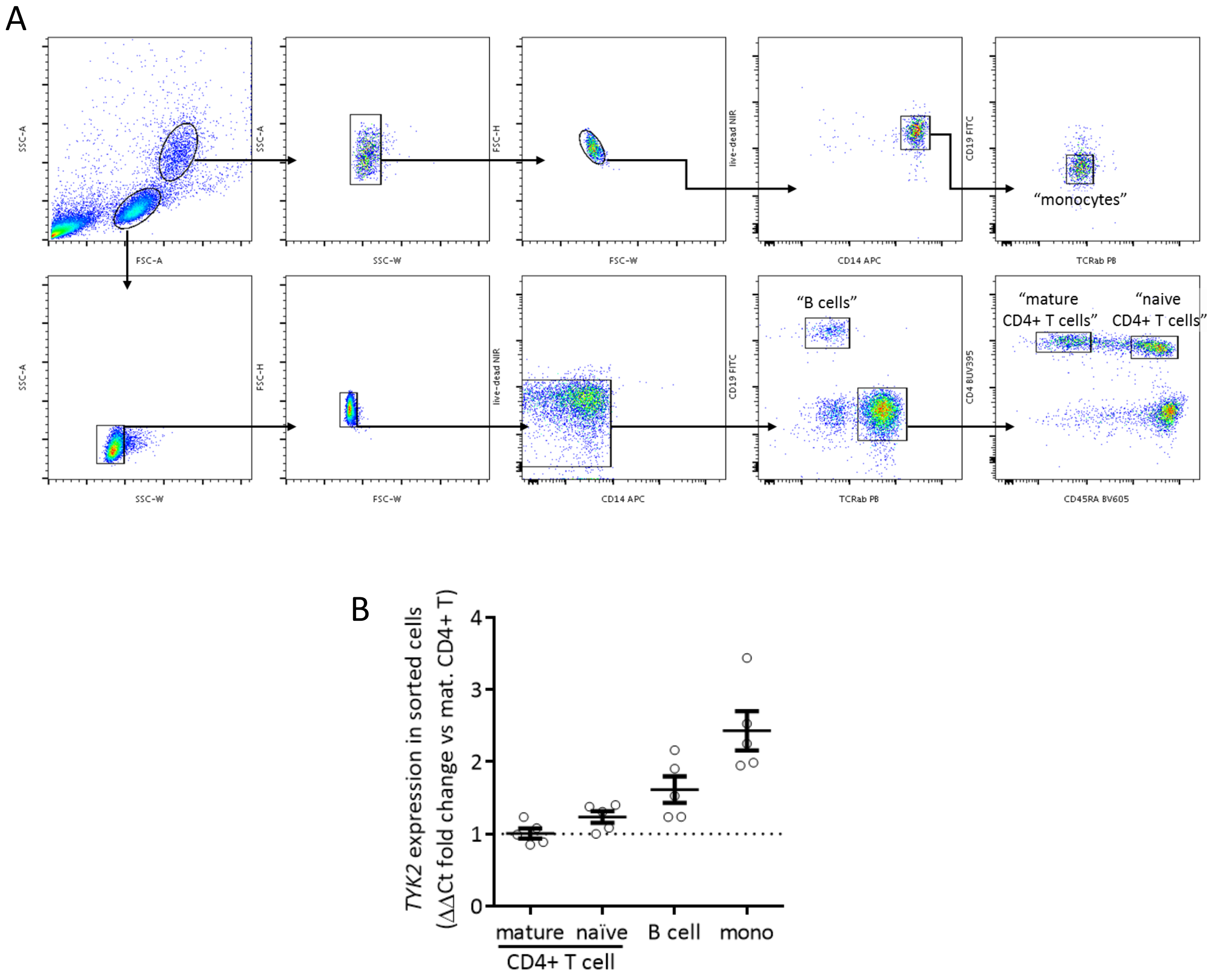

Supplementary Figure 12: *TYK2* expression in FACS sorted cells. A) Gating strategy for cell sorting to validate *TYK2* expression at the cellular level by qPCR. B) TYK2 expression in sorted cells from 5 subjects assessed by qPCR and normalized to *HPRT.* Each datapoint is an independent donor.

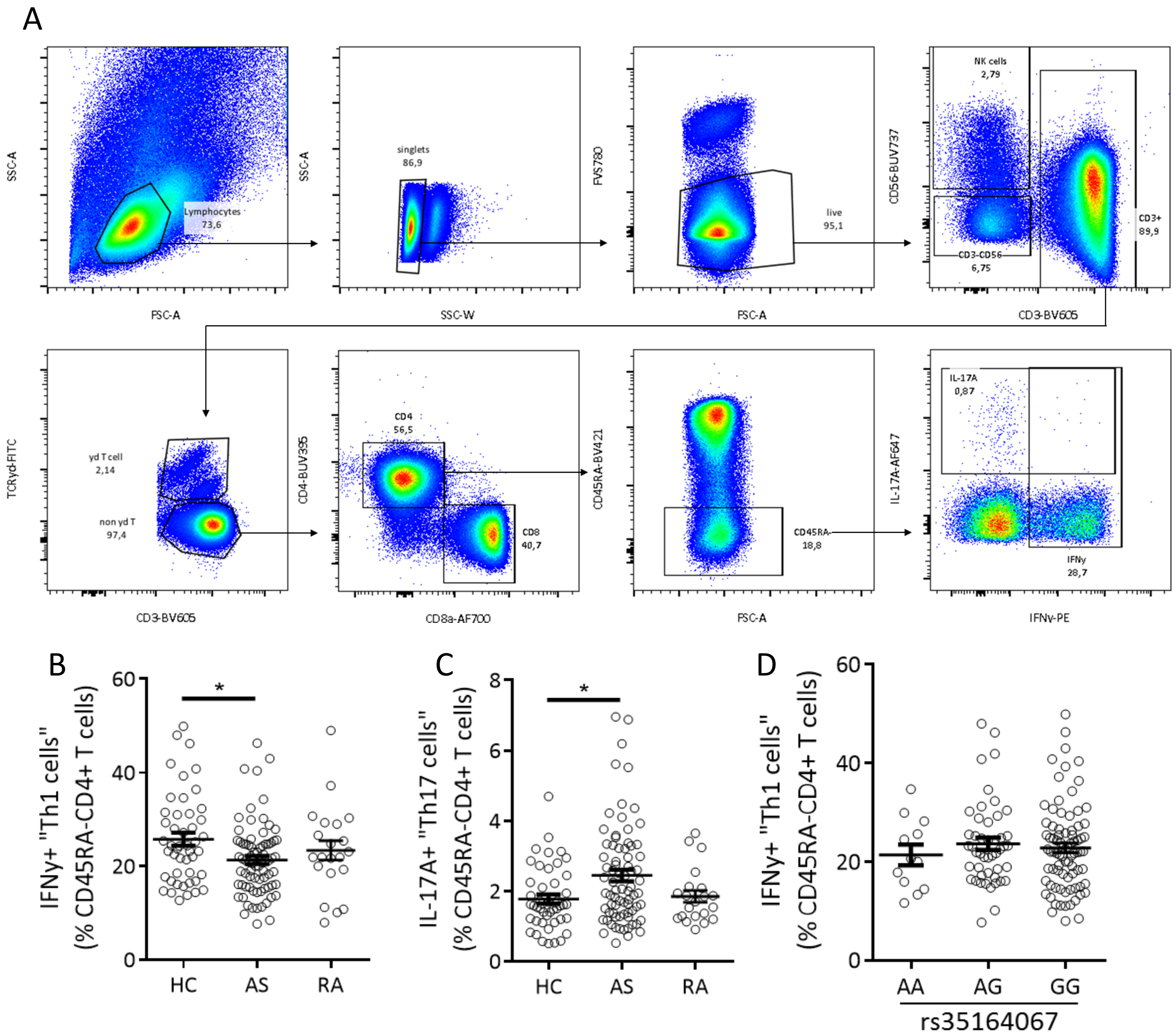

Supplementary Figure 13: Extended FACS data of PMA/ionomycin stimulated PBMC. A) Gating strategy for the identification of NK cells, IFNγ+ (Th1) and IL-17A+ (Th17) mature CD4+ T cells. Th1 (B) and Th17 (C) frequency in cohort. D) Th1 frequency in pooled AS/RA/HC subjects stratified by the intergenic *TYK2* SNP, rs35164067. Data analyzed by Mann-Whitney test. Each datapoint is an independent donor.

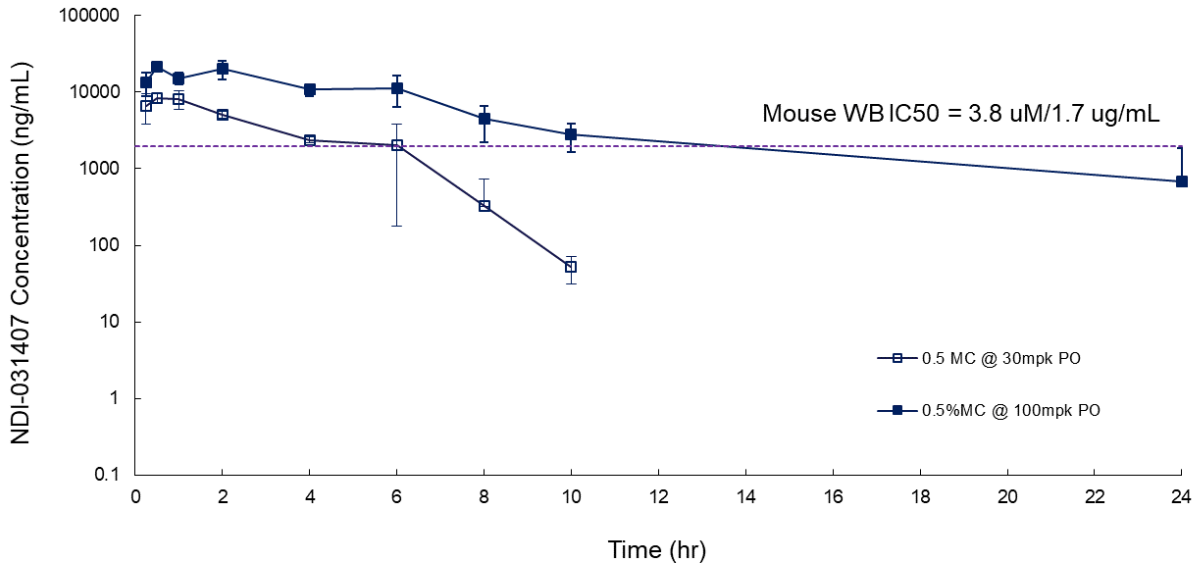

Supplementary Figure 14: Mouse PK time course for NDI-031407. Dotted line is the IC50 of NDI-031407 in IL-12 induced IFNγ assay with mouse whole blood. PK study was conducted in C57BL/6 mice dosed by oral gavage with NDI-031407 in 0.5% methylcellulose. Plasma was prepared from blood drawn at indicated time points and analyzed for NDI-031407 concentration using LC/MS. Mouse whole blood assay was performed by incubating the compound with blood for one hour followed by IL-12 stimulation for 24 hours on anti-CD3 antibody coated plate. At the end of the incubation, IFNγ level in the supernatant was quantified by mesoscale discovery assay (not shown).
